# Establishment of a tobacco BY2 cell line devoid of plant‐specific xylose and fucose as a platform for the production of biotherapeutic proteins

**DOI:** 10.1111/pbi.12702

**Published:** 2017-03-03

**Authors:** Uri Hanania, Tami Ariel, Yoram Tekoah, Liat Fux, Maor Sheva, Yehuda Gubbay, Mara Weiss, Dina Oz, Yaniv Azulay, Albina Turbovski, Yehava Forster, Yoseph Shaaltiel

**Affiliations:** ^1^ Protalix Biotherapeutics Carmiel Israel

**Keywords:** glyco‐engineering, bio‐pharming, DNaseI, plant glycans, CRISPR/Cas9, gene editing

## Abstract

Plant‐produced glycoproteins contain N‐linked glycans with plant‐specific residues of β(1,2)‐xylose and core α(1,3)‐fucose, which do not exist in mammalian‐derived proteins. Although our experience with two enzymes that are used for enzyme replacement therapy does not indicate that the plant sugar residues have deleterious effects, we made a conscious decision to eliminate these moieties from plant‐expressed proteins. We knocked out the β(1,2)‐xylosyltranferase (*XylT*) and the α(1,3)‐*fucosyltransferase* (*FucT*) genes, using CRISPR/Cas9 genome editing, in *Nicotiana tabacum* L. cv Bright Yellow 2 (BY2) cell suspension. In total, we knocked out 14 loci. The knocked‐out lines were stable, viable and exhibited a typical BY2 growing rate. Glycan analysis of the endogenous proteins of these lines exhibited N‐linked glycans lacking β(1,2)‐xylose and/or α(1,3)‐fucose. The knocked‐out lines were further transformed successfully with recombinant DNaseI. The expression level and the activity of the recombinant protein were similar to that of the protein produced in the wild‐type BY2 cells. The recombinant DNaseI was shown to be totally free from any xylose and/or fucose residues. The glyco‐engineered BY2 lines provide a valuable platform for producing potent biopharmaceutical products. Furthermore, these results demonstrate the power of the CRISPR/Cas9 technology for multiplex gene editing in BY2 cells.

## Introduction

The use of plant cell suspension culture as a host for the production of recombinant proteins is gaining more and more popularity (Santos *et al*., 2016) and the *Nicotiana tabacum* (*N. tabacum*) cv. Bright yellow 2 (BY2) cell line is among the most commonly used cells for this purpose (Mercx *et al*., 2016). Plant‐produced proteins contain an N‐linked glycan core structure similar to what is found in mammalian cells, but they may also include additional core α(1,3)‐fucose and β(1,2)‐xylose, not found in mammalian‐produced proteins (Tekoah *et al*., 2015).

While there is no clinical evidence indicating differences in immunogenic response to plant‐specific glycans versus mammalian‐derived glycans (Santos *et al*., 2016; Tekoah and Shaaltiel, 2016), extensive research efforts have been undertaken in recent years to modulate the plant‐specific N‐glycosylation machinery aiming at the production of recombinant proteins with mammalian‐like modifications.

The transfer and the attachment of plant‐specific sugar moieties to the developing glycan structure are carried out by the two Golgi resident enzymes α(1,3)‐*fucosyltransferase* (*FucT*) and β(1,2)‐*xylosyltransferase* (*XylT*) (Strasser *et al*., 2004). Various strategies such as RNA interference (RNAi) technology and random mutagenesis methods have been applied, in various plant species, to interfere with the function of the *FucT* and *XylT* genes (Castilho *et al*., 2011; Cox *et al*., 2006; Strasser *et al*., 2008; Weterings and Gerben, 2013; Yin *et al*., 2011). Other genes, involved in the plant‐specific N‐glycosylation pathway, were also addressed. RNAi methodology was used to modulate the *GMD* gene, encoding the Guanosine 5′‐diphosphate (GDP)‐D‐mannose 4,6‐dehydratase enzyme, which is associated with GDP‐L‐fucose biosynthesis in *Nicotiana benthamiana* (*N. benthamiana*) plants. This resulted in N‐glycans with decreased α(1,3)‐fucose residues (Matsumura, 2010).

Stable expression of *GnTI* (*N*‐acetylglucosaminyltransferase I) antisense in potato (*Solanum tuberosum*) and in tobacco (*N. tabacum*) showed no significant changes in the total N‐glycan profiling versus the wild‐type plants (Wenderoth and von Schaewen, 2000), while the down‐regulation of the *GnTI* in *N. benthamiana*, using RNAi, resulted in incomplete elimination of the plant‐specific glycans (Limkul *et al*., 2016). Apparently none of the above approaches were able to completely deactivate the functions of the targeted genes, especially when more than one gene or a gene family was involved.

An alternative approach for the production of recombinant glycoproteins lacking xylose and fucose residues is to knock out the *β(1,2)‐xylosyltransferase* (*XylT*) and *α(1,3)‐fucosyltransferase* (*FucT*) genes using targeted genome editing. Targeted genome editing using zinc finger nucleases (ZFNs; Kumar *et al*., 2006), transcription activator‐like effector nucleases (TALENs; Christian *et al*., 2010), or the recently developed CRISPR/Cas9 (Clustered Regularly Interspaced Short Palindromic Repeats) technology (Cong *et al*., 2013; Jinek *et al*., 2012) has been achieved by their ability to cleave DNA and create double‐strand breaks (DSBs) at specific sites in the genome (Gaj *et al*., 2013). DSBs at a gene target site are most often repaired by the nonhomologous end joining (NHEJ) DNA repair mechanism. This repair is often accompanied by small insertions or deletions (in‐del) of nucleotides at the site of repair which can cause a mutation and knock out the targeted gene (Gorbunova and Levy, 1999).

Recently, the TALEN‐mediated targeted gene editing technology was employed to manipulate N‐glycosylation pathways in *N. benthamiana* (Li *et al*., 2016). The endogenous proteins of the knocked‐out line had N‐glycans that lacked β(1,2)‐xylose but demonstrated only a 40% reduction in core α(1,3)‐fucose levels when compared to the proteins from the wild‐type cells. The authors suggested that the residual activity of the *FucT* enzyme in the mutated line might be explained by the presence of multiple copies of the *FucT* genes in the *N. benthamiana* genome.

The CRISPR/Cas9 technology comprises small guide RNAs (gRNA), which identify and locate the targeted DNA sequence, and an associate DNA endonuclease (Cas9), which execute the sequence‐specific cleavage (Jinek *et al*., 2012). Two types of gRNAs—designated as CRISPR RNA (crRNA or the ‘protospacer’) and *trans*‐acting RNA (tracrRNA)—are known to be involved in the guiding process. The crRNA guides the Cas9 endonuclease to the specific DNA target, while the actual binding of the enzyme to the DNA is mediated by the tracrRNA (Jinek *et al*., 2012). The DNA sequence encoding the crRNA is about 20 nucleotides long and is terminated, at its 3′ end, with a 3‐base‐pair proto spacer adjacent motif (PAM) of the sequence NGG (N can be any nucleotide) required for the correct recognition of the target. To facilitate the practical application of the system, the two separate gRNAs were assembled into a single chimeric molecule—designated single guide RNA (sgRNA; Jinek *et al*., 2012). Because of their small size, multiple sgRNAs can be co‐constructed with the Cas9 on the same vector to achieve ‘multiplex gene editing’ (Belhaj *et al*., 2013).

The *N. tabacum* genome is known to contain two *XylT* genes (Ntab‐*XylT*) and five *FucT* genes (Ntab‐*FucT*; Table 1). Thus, to achieve *N. tabacum* BY2 cells devoid of any activity of these enzymes, simultaneous editing of seven genes and two alleles per gene is needed. Based on the results recently achieved with the CRISPR/Cas9 technology in accomplishing targeted DNA modifications in a wide variety of organisms (Cong *et al*., 2013; Mali *et al*., 2013) including several plant species (Brooks *et al*., 2014; Jacobs *et al*., 2015; Jia and Wang, 2014; Jiang *et al*., 2013, 2014; Mercx *et al*., 2016; Schiml and Puchta, 2016), this technology was chosen to knock out the *XylT* and the *FucT* genes.

**Table 1:** Details of the *N. tabacum XylT* and *FucT* genes

Gene family	Name	Accession numbers^a^	Remarks
XylT	Ntab‐*XylT*‐A	Ntab‐BX_AWOK‐SS596	Alignment analysis of the open reading frame showed 94% identity between the two genes
	Ntab‐*XylT*‐B	Ntab‐BX_AWOK‐SS12784	
FucT	Ntab‐*FucT‐A*	Ntab‐K326_AWOJ‐SS19752	These three *FucT* genes were grouped into ‘Group 1’ based on the high percentage of identity between the nucleotides sequence of their first and second exons
	Ntab‐*FucT‐B*	Ntab‐BX_AWOK‐SS16887	
	Ntab‐*FucT‐C*	Ntab‐K326_AWOJ‐SS16744	
	Ntab‐*FucT‐D*	Ntab‐K326_AWOJ‐SS19661	These two *FucT* genes were grouped into ‘Group 2’ based on the 98% identity between their third exons
	Ntab‐*FucT‐E*	Ntab‐K326_AWOJ‐SS19849	

a The Sol Genomic Network (www.solgenomic.net).

The goal of this project was to completely eliminate the β(1,2)‐xylose and α(1,3)‐fucose plant‐specific glycans in the Protalix's BY2 cell system, enabling the production of recombinant glycoproteins lacking these sugar moieties.

## Results

### Isolation of the targeted genes in the BY2 cells

Based on the published sequences of the Ntab‐*XylT*‐A and Ntab‐*XylT*‐B (Table 1), two primers were designed (Table S1, items 1, 2) and were used in a polymerase chain reaction (PCR) to isolate a partial sequence of *XylT* genes from the BY2 cells. The reaction revealed two PCR products. The first 2161‐bp product was identical to a fragment of the published Ntab‐*XylT*‐A sequence and was labelled BY2‐*XylT*‐A (Figure S1). The second 2145‐bp product was identical to a fragment of the published Ntab‐*XylT*‐B sequence and was labelled BY2‐*XylT*‐B (Figure S2).

**Figure 1:**
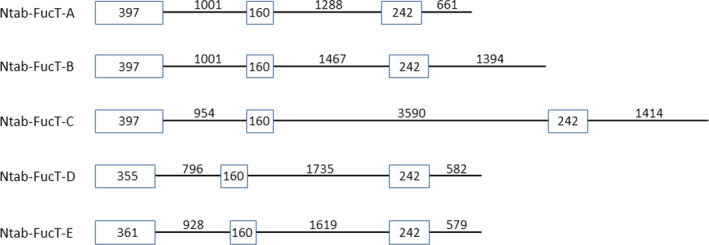
Schematic illustration of the first three exons and introns of the five *FucT* genes of *N. Tabaccum*. Exons 1, 2, 3 are shown as boxes and introns 1, 2, 3 as lines. The size of each exon and intron is indicated in base pairs (bp).

Five Ntab‐*FucT* genes are publicly known (Table 1). Based on an alignment analysis of these five variants and for the purpose of this work, we clustered the five genes into two groups. The first group contained the Ntab‐*FucT*‐*A*, Ntab‐*FucT*‐*B* and Ntab‐*FucT*‐*C* genes that share 96% of identity between the sequences of their first three exons (Table 1; Figure 1). The second group included the Ntab‐*FucT*‐*D* and Ntab‐*FucT*‐*E* genes that share 98% identity between their third exons (Table 1; Figure 1). Accordingly, two different sets of primers were designed (Table S1, items 3–6) and were used in a PCR to isolate parts of the sequence of each of the five *FucT* genes from the BY2 cells genome.

Using the first pair of primers (Table S1, items 3,4) resulted in two PCR products: a 3089‐bp PCR product, showing 99.9% identity with exons 1,2,3 and introns 1 and 2 of both the Ntab‐*FucT*‐*A* and Ntab‐*FucT*‐*B* genes and a 5343‐bp PCR product sharing 99.9% identity with exons 1,2,3 and introns 1 and 2 of the Ntab‐*FucT‐C* gene. Based on the identity between the first three exons of the Ntab‐*FucT*‐*A* and Ntab‐*FucT*‐*B* genes, the first PCR product can correspond to either gene and therefore was referred to as BY2‐*FucT*‐A and BY2‐*FucT*‐B (Figure S3). The second product was referred as BY2‐*FucT‐C* (Figure S4).

Using the second pair of primers (Table S1, items 5, 6) resulted in another two DNA fragments: a 834‐bp product identical to the sequence of the final part of intron 2, exon 3 and the initial part of intron 3 of the Ntab‐*FucT‐D* gene and a 832‐bp product identical to the final part of intron 2, exon 3 and the initial part of intron 3 of the N.tab‐*FucT*‐E gene. These fragments were labelled BY2‐*FucT*‐D (Figure S5) and BY2‐*FucT*‐E (Figure S6), respectively.

### Construction of the Cas9/sgRNA constructs to knock out the XylT and the FucT genes

Following the identification of the BY2‐*XylT* and the BY2‐*FucT* genes in the BY2 cells, various DNA sequences, all starting with nucleotide G and tailed with the required PAM at their 3′ ends, were selected as the Cas9 targets. Accordingly, the following five crRNAs were defined (Table S2): **crRNA1**—a 20‐bp DNA sequence shared between the BY2‐*XylT*‐A and the BY2‐*XylT*‐B genes (Table S2, item 1); **crRNA2** and **crRNA3**, 18 and 21 bp long, respectively, were chosen based on DNA sequences shared between the BY2‐*FucT*‐A, B and C genes (Table S2, items 2,3); **crRNA4** and **crRNA5**, each 20 bp long, were chosen based on two DNA sequences shared between the BY2‐*FucT*‐D and the BY2‐*FucT*‐E (Table S2, items 4,5).

For the application of the CRISPR/Cas9 technology, the five crRNAs were each fused to the tracrRNA backbone sequence (Figure S7) resulting in the construction of five sgRNAs (designated sgRNA1—sgRNA5).

**Figure 2:**
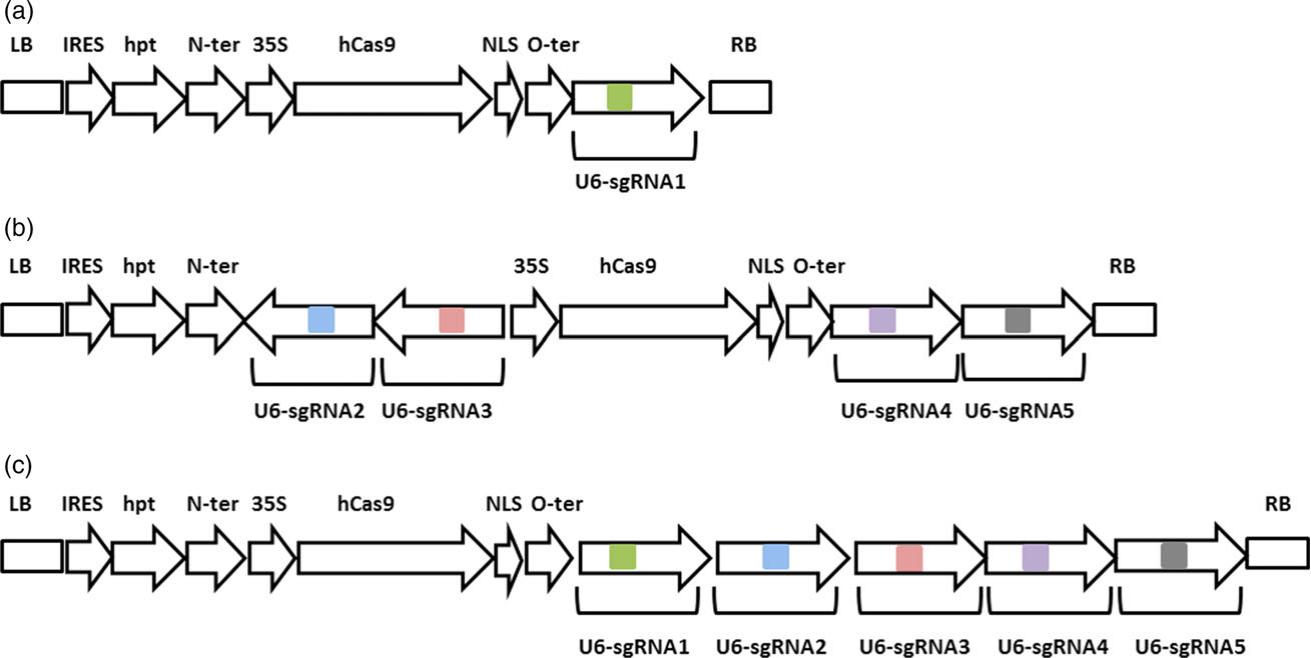
Schematic description of the three binary vectors used for the transformation of the BY2 cells. (a) The phCas9‐XylT; (b) the phCas9‐FucT; (c) the phCas9‐XylT/FucT. LB, left border; IRES, internal ribosome entry site; hpt, hygromycin phosphotransferase; N‐ter, nopaline synthase terminator; 35S, 35S cauliflower mosaic virus promoter with omega enhancer; hCas9, human‐optimized Cas9; NLS, SV40 nuclear localization signal; O‐ter, octopine synthase terminator; U6, Arabidopsis U6 promoter; sgRNA, chimeras of the various crRNAs with tracrRNA, the colour boxes represent the five different crRNAs that were used; RB, right border.

Three binary vectors namely **phCas9‐XylT** (Figure 2a), **phCas9‐FucT** (Figure 2b) and **phCas9‐XylT‐FucT** (Figure 2c) were then constructed and used in three separate cell transformations aiming at the knockout of either the BY2‐*XylT* genes, the BY2‐*FucT* genes or both groups of genes within the same cell.

The **phCas9‐XylT** vector (Figure 2a) comprised three cassettes: a *Hygromycin phophotransferase (hpt)* selectable marker under an internal ribosome entry site (IRES) sequence, followed by a human‐optimized Cas9 (Nekrasov *et al*., 2013) attached to a nuclear localization signal (NLS) at its 3′ terminal end, driven by 35S cauliflower mosaic virus promoter (35S) and the sgRNA1 under the Arabidopsis U6 promoter.

The **phCas9‐FucT** (Figure 2b) contained the following six cassettes: *hpt* selectable marker under the IRES; a human‐optimized synthetic Cas9 (Nekrasov *et al*., 2013) attached to a nuclear localization signal (NLS) at its 3′ terminal end, driven by 35S promoter; and four sgRNA cassettes, each under the Arabidopsis U6 promoter, and each containing one of the gRNA2 through gRNA5 fused to a tracrRNA.

The **phCas9‐XylT‐FucT** (Figure 2c) contained the following seven cassettes: a *hpt* selectable marker under IRES; a human‐optimized synthetic Cas9 (Nekrasov *et al*., 2013) attached to a nuclear localization signal (NLS) at its 3′ terminal end, driven by 35S promoter; and all the five sgRNAs that were previously described and used in this project, each under the Arabidopsis U6 promoter.

### Knocking out of the XylT and the FucT genes in the BY2 cells

The three constructed vectors were used in three separate stable transformations of BY2 cells. In the first transformation, aiming at the knockout of the BY2‐*XylT* genes and using the phCas9‐XylT (Figure 2a), a total of 110 individual cell lines were isolated and screened. Total soluble protein was extracted from the cells and separated on SDS‐PAGE, followed by transfer to a nitrocellulose membrane and detection by Western blot analysis using anti‐xylose antibodies. About 30% of the screened lines were found negative to the anti‐xylose antibody.

In the second transformation, aiming at the knockout of the BY2‐*FucT* genes and using the phCas9‐FucT (Figure 2b) about 100 individual cell lines were isolated and screened. Total soluble protein was extracted from the cells and separated on SDS‐PAGE, followed by a Western blot analysis using anti‐fucose antibodies. About 60% of the screened lines were found negative to the anti‐fucose antibody.

In the third transformation, using the phCas9‐XylT‐FucT vector (Figure 2c) and aiming at knocking out both groups of genes (the *XylT* and the *FucT*), a total of 250 individual lines were isolated and screened sequentially for the absence of fucose and xylose. Screening for fucose‐free lines was conducted by using an ELISA test. Total soluble protein from the putative fucose‐free lines (about 30% of the screened lines) was separated by SDS‐PAGE followed by a Western blot analysis using anti‐xylose. Twenty five (10% of the total 250 lines) lines were assumed to be knocked out for both *XylT* and *FucT*.

**Figure 3:**
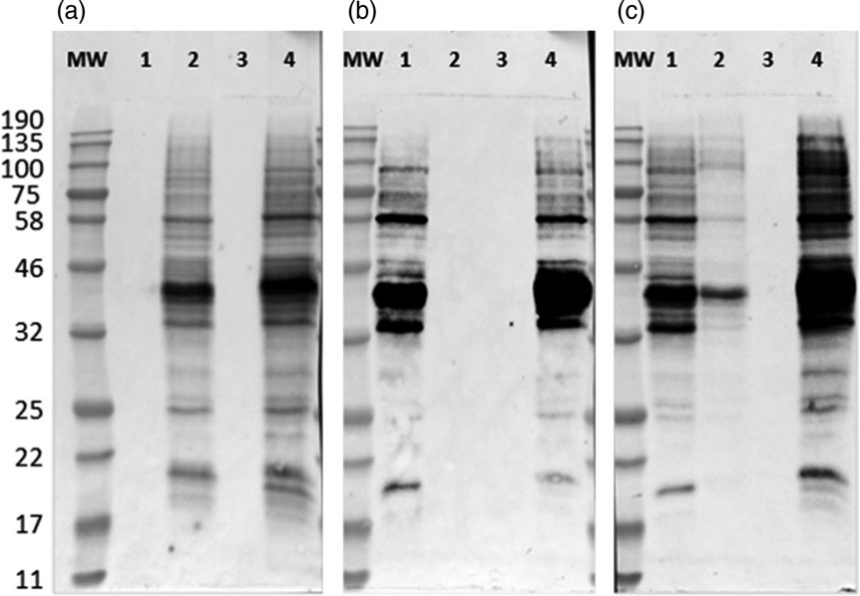
Western blots using anti‐xylose, anti‐fucose or anti‐HRP antibodies. The total soluble protein was extracted from the glyco‐engineered and the nontransgenic BY2 cells and 10 μg of protein from each sample were loaded on SDS‐PAGE followed by Western blot using (a) anti‐xylose, (b) anti‐fucose and (c) anti‐HRP antibodies. 1, ΔXT cell line; 2, ΔFT cell line; 3, ΔXFT cell line; 4, the wild‐type nontransgenic BY2 cells. MW, molecular weight marker in kDa.

Western blots of the total soluble protein extracted from three selected glyco‐engineered cell lines and the nontransgenic BY2 cells, using anti‐xylose, anti‐fucose or anti‐HRP antibodies, are presented in Figures 3 and S8.

### Identification and characterization of the mutations generated by the Cas9 multiplexed targeting of the XylT and the FucT genes

The mutations that were generated by the Cas9 were characterized in three different mutated cell lines, that is in a *XylT* knocked‐out cell line (ΔXT), in a *FucT* knocked‐out cell line (ΔFT) and in a double knocked‐out cell line (ΔXFT).

For each of the three cell lines, a PCR was performed using primers flanking the Cas9 target sites (Table S3). The obtained PCR products were cloned into a pGEMT vector and 36–60 clones for each sample were sequenced revealing the presence of assorted insertions and deletions (in‐dels). No wild‐type products were detected among any of the tested clones.

Three types of mutations were demonstrated for the ΔXT cell line: an identical 36‐bp deletion in both alleles of *XylT*‐A and a 1‐bp insertion and a 13‐bp deletion in *XylT*‐B (Figure S9).

Seven assorted in‐dels mutations were identified in the ΔFT cell line: an identical 1213‐bp deletion in the *FucT*‐A; an identical 2‐bp insertion in the *FucT*‐B; a 7‐bp and a 15‐bp deletions in the *FucT*‐C; a 3‐bp and a 7‐bp deletions in the *FucT*‐D and an identical 5‐bp deletion in the *FucT*‐E (Figure S10).

In the ΔXFT cell line, three mutations were identified for the *XylT* genes and ten for the *FucT* genes (Figure S11). An identical 7‐bp deletion was found in both alleles of the *XylT‐*A; a 1‐bp and a 13‐bp deletions were identified in the *XylT*‐B. A 7‐bp deletion and a 2‐bp insertion were found in *FucT*‐A; a 22‐bp and a 21‐bp deletions in *FucT*‐B; a 7‐bp and a 47‐bp deletions were demonstrated in *FucT*‐C; a 72‐bp and a 37‐bp deletions in *FucT*‐D and a 56‐bp insertion and a 37‐bp deletion were found in *FucT*‐E.

### Glycan analysis of proteins derived from the XylT, the FucT and the XylT/FucT knocked‐out lines

**Figure 4:**
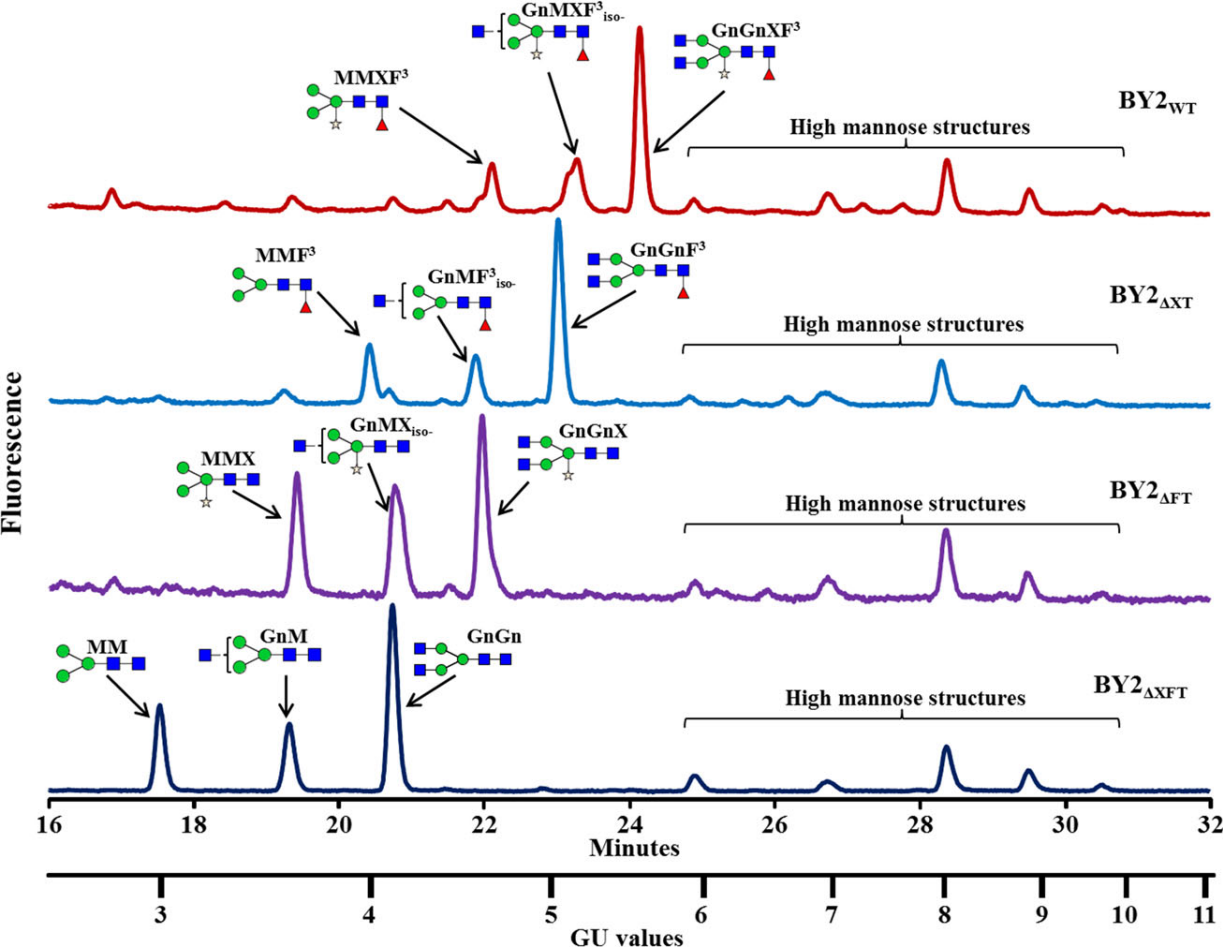
UPLC chromatograms presenting the peaks of the main glycans derived from the glycoproteins derived from (top to bottom): BY2 control cells (WT); a *XylT* knocked‐out line (ΔXT); a *FucT* knocked‐out line (ΔFT); and a *XylT/FucT* knocked‐out cell line (ΔXFT). Glycans are annotated with cartoons and acronyms above main peaks. Symbols are as follows: 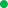
‐Mannose; 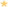
‐Xylose; 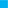
‐*N*‐acetylglucosamine; 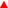
‐Fucose. Glycan acronyms are based on the nomenclature indicated at the following website http://www.proglycan.com/sites/default/public/pdf/nomen_2007.pdf.

Glycan analysis of the total soluble proteins extracted from the three various knocked‐out cell lines (ΔXT—Δ*XylT* cell line, ΔFT—Δ*FucT* cell line and ΔXFT—Δ*XylT*/Δ*FucT* cell line) was compared with the glycans found in the wild‐type BY2 control cells. The glycan pools, separated on a normal phase UPLC system coupled with a fluorescence detector, showed a clear shift to shorter retention times [smaller glucose unit (GU) values] in the three mutated cell lines (Figure 4) compared to the control sample (Figure 4, top panel). The main glycan peaks were identified and are annotated on the top of each glycan profile. The samples of the released glycan pools were then subjected to digestion by various exoglycosidases and analysis by matrix‐assisted laser desorption ionization (MALDI) time‐of‐flight (ToF) mass spectrometry (MS), confirming the glycan pool assignments of the total soluble proteins from the various cell lines. Results from exoglycosidase digestion and MS from the double‐mutated BY2 cell line (ΔXFT) are presented in Figures S12 and S13.

The prevalence of xylose, fucose and xylose‐/fucose‐containing glycans among the total glycans derived from each of the tested samples is summarized in Table 2. Whereas 80% and 77% of the various glycans that were released from the proteins derived from the wild‐type BY2 cells contained either xylose or fucose residues, respectively, the glycans that were released from the proteins derived from the ΔXT cell lines contained no xylose; those released from the ΔFT cell lines contained no fucose; and neither xylose nor fucose were identified in the glycans that were released from the proteins derived from the ΔXFT cell lines. Additional glycans, identified as high mannose structures, did not change between the various cell lines, as expected, because they did not include any fucose or xylose sugars. Exoglycosidase digestion and MALDI‐ToF mass spectrometry data of the ΔXFT cell lines verify that the predominant glycans are of the paucimannose type (MM) or paucimannose, substituted with either one or two *N*‐acetylglycosamine sugars on the terminal mannose (GnM and GnGn, respectively). In addition, a variety of high mannose glycans (Man6‐Man9) can be found. All glycans did not contain xylose or fucose.

**Table 2:** Distribution of the glycans in the various BY2 cell lines

Glucose unit value	Glycan acronym^a^	WT	∆XT	∆FT	∆XFT
4.4	MM	–	–	–	27^b^
4.9	GnM	–	4	–	13
4.9	MMX	3	–	21	–
5.3	MMF^3^	–	14	–	–
5.3	GnMX	–	–	25	–
5.4	Man4	4	5	–	–
5.5	GnGn	–	–	–	34
5.8	GnGnX	–	–	32	–
5.9	GnMF^3^	–	13	–	–
5.9	MMXF^3^	17	–	–	–
6.3	GnGnF^3^	–	43	–	–
6.3	GnMXF^3^	22	–	–	–
6.8	GnGnXF^3^	38	–	–	–
7.1	Man6	3	–	2	5
8.0	Man7	4	4	4	4
8.9	Man8	7	12	11	11
9.6	Man9	3	5	4	6

a Based on nomenclature from http://www.proglycan.com/sites/default/public/pdf/nomen_2007.pdf.

b Results presented are of glycans above 2% of total pool.

### Glycan analysis of recombinant DNaseI expressed in the XylT, FucT and XylT/FucT knocked‐out cell lines

**Figure 5:**
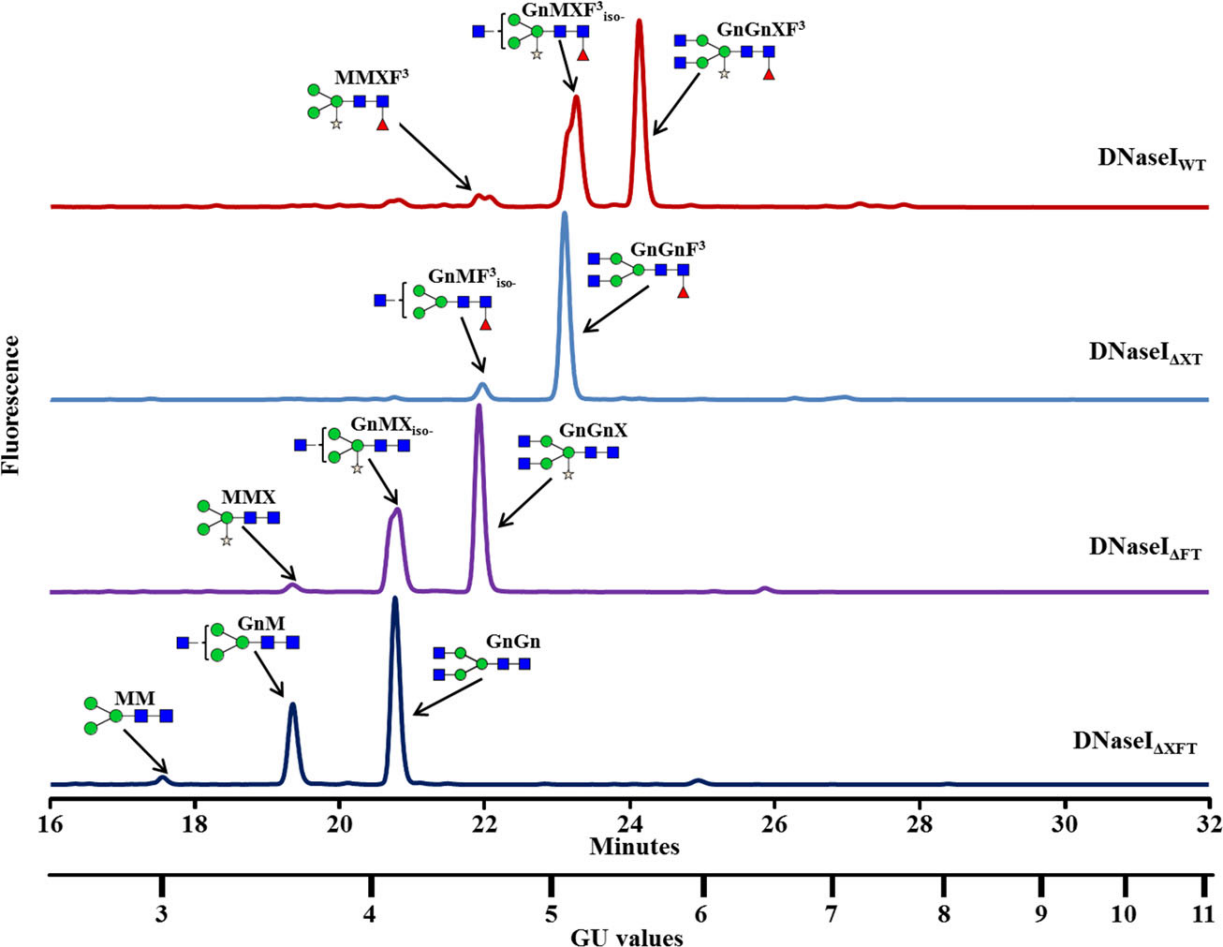
UPLC chromatograms presenting the peaks of the main glycans released from a DNaseI protein (top to bottom): Glycan pool derived from DNaseI produced by control cells (WT); glycan pool from a *XylT* knocked‐out line (ΔXT); glycan pool released from DNaseI produced from a *FucT* knocked‐out line (ΔFT); and a glycan pool released from DNaseI from a *XylT/FucT* knocked‐out cell line (ΔXFT). Glycans are annotated with cartoons and acronyms above main peaks. Symbols are as follows: 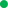
‐Mannose; 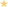
‐Xylose; 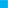
‐*N*‐acetylglucosamine; 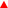
‐Fucose. Glycan acronyms are based on the nomenclature indicated at the following website http://www.proglycan.com/sites/default/public/pdf/nomen_2007.pdf.

To demonstrate that the knocked‐out cell lines can be used to produce recombinant proteins that do not contain plant‐specific sugar moieties, the glyco‐engineered cells were transformed with human recombinant DNaseI (Figure S14) and compared with the control BY2 cells that underwent the same transformation. The various cell lines underwent transformation at typical rates and expressed comparable amounts of total and active DNaseI (0.92 ± 0.11; 0.97 ± 0.03; 0.91 ± 0.07; 0.89 ± 0.08 μg active DNAseI per μg of total DNaseI for the WT BY2 cells, the ΔXT, the ΔFT and the ΔXFT cells, respectively), measured in crude extract. The recombinant DNaseI was purified from the culture medium, and the specific activity was evaluated using a methyl green‐based activity assay at 37 °C. Results show that all variants were equally active, with specific activity values of 4.1 ± 0.2, 3.9 ± 0.1, 4.4 ± 0.1 and 4.1 ± 0.3 μg DNA/min/μg DNase for the WT BY2 cells, the ΔXT, the ΔFT and the ΔXFT cells, respectively. The various purified DNaseI were run on SDS‐PAGE, and the glycans were separated from the isolated protein band on the gel. The glycans derived from the recombinant DNaseI produced in the glyco‐engineered cell lines and in the control BY2 cells were then separated on UPLC (Figure 5). The results showed a significant difference between the glycan profiles of the various samples. While the glycans derived from the DNaseI produced in the control BY2 WT line (DNaseI_WT_) all contained xylose and fucose (Figure 5, top panel), the glycans derived from the DNaseI produced by the ΔXT cells, by the ΔFT cells and by the ΔXFT cells (DNaseI_ΔXT_, DNaseI_ΔFT_, DNaseI_ΔXFT_, respectively) contained either no xylose, no fucose or neither xylose nor fucose (Figure 5, three lower panels), respectively. A summary of the various glycans is presented in Table 3. Exoglycosidase digestion and MALDI‐ToF mass spectrometry data (S15 and S16, respectively) verify that the predominant glycans of the DNaseI_ΔXFT_ protein are of the paucimannose type (MM) or paucimannose, substituted with either one or two *N*‐acetylglycosamine sugars on the terminal mannose (GnM and GnGn, respectively). The DNaseI glycan did not contain any high mannose glycans (Man6‐Man9) as expected from a secreted protein.

**Table 3:** Distribution of the glycans in the various produced DNase I products

Glucose unit value	Glycan acronym^a^	WT	∆XT	∆FT	∆XFT
4.4	MM	–	–	–	3^b^
4.9	GnM	–	–	–	30
4.9	MMX	–	–	3	–
5.3	MMF^3^	–	–	–	–
5.3	GnMX	–	–	40	–
5.4	Man4	–	–	–	–
5.5	GnGn	–	–	–	67
5.8	GnGnX	–	–	56	–
5.9	GnMF^3^	–	8	–	–
5.9	MMXF^3^	4	–	–	–
6.3	GnGnF^3^	–	92	–	–
6.3	GnMXF^3^	45	–	–	–
6.8	GnGnXF^3^	51	–	–	–
7.1	Man6	–	–	–	–
8.0	Man7	–	–	–	–
8.9	Man8	–	–	–	–
9.6	Man9	–	–	–	–

a Based on nomenclature from http://www.proglycan.com/sites/default/public/pdf/nomen_2007.pdf.

b Results presented are of glycans above 2% of total pool.

The DNaseI_BY2_ had two major peaks (Figure 5 top)—a peak identified as a bianntenary core‐fucosylated and xylosylated glycan (GnGnXF^3^, 51%) and a monoanntenary core‐fucosylated and xylosylated peak (GnMXF^3^, 45%). A third peak (4%) was identified as a paucimannose structure substituted with xylose and fucose (MMXF^3^). In the DNaseI_ΔXT_, the main peak (92%) was assigned as a bianntenary core‐fucosylated glycan (GnGnF^3^) and the minor peak (8%) as a monoanntenary core‐fucosylated glycan (GnMF^3^; Figure 5, DNaseI_ΔXT_). In the DNaseI_ΔFT_, the main peak (56%) was assigned as a bianntenary glycan with xylose (GnGnX) and the minor peaks (40%, 3%) as a monoanntenary glycan containing xylose (GnMX) or a paucimannose structure substituted with a bisecting xylose (MMX), respectively (Figure 5, DNaseI_ΔFT_). The DNaseI_ΔXFT_ main peak (67%) was assigned as a nonfucosylated/nonxylosylated bianntenary glycan (GnGn), and its minor peaks (30% and 3%) were identified as a nonfucosylated/nonxylosylated monoanntenary glycan (GnM) or a paucimannose structure (MM; Figure 5, DNaseI_ΔXFT_).

## Discussion

Plants and plant cell suspensions in particular are considered as a promising platform for the production of biopharmaceuticals proteins (Santos *et al*., 2016). Furthermore, plant cell suspension was the first plant‐based system that produced an ERT product approved by the FDA and other authorities around the world (Fox, 2012).

Aiming at eliminating the plant‐specific glycans β(1,2)‐xylose and α(1,3)‐fucose from recombinant glycoproteins that are produced in *N. tabacum* BY2 cells, we utilized the CRISPR/Cas9 technology to produce various cell lines devoid of these specific plant glycans. Three BY2 glyco‐engineered cell lines were established: *XylT*,* FucT* and both *XylT* and *FucT* knocked‐out lines. All three lines demonstrated complete eradication of the targeted glycans, respectively. The knocked‐out cell lines were stable, viable and exhibited a typical BY2 growing rate. As the presence of glycans β(1,2)‐xylose and α(1,3)‐fucose in glycoprotein is conserved in plants, the absence of an apparent difference between the WT and the knockout cell lines should be further studied. It might be that fucosylation and xylosylation of plant glycoproteins play a role under certain stress conditions, or during development *in planta*. In this respect, we show here, for the first time, that the presence of xylose or fucose sugars in glycoproteins is not vital for the growth of BY2 cells in culture.

We were then able to utilize the new cell lines to produce a specific biotherapeutic protein. A subsequent transformation of the mutated lines with recombinant DNaseI showed that the biotherapeutic did not contain the β(1,2)‐xylose and/or the α(1,3)‐fucose in its glycans. This was used as a model to show that any recombinant glycoprotein can be produced in Protalix's glyco‐engineered BY2 cells.

This study has added the *N. tabacum* cv. BY2 cells to the list of plant systems that were shown to be amenable to targeted DNA modifications by applying the CRISPR/Cas9 technology. Furthermore, by simultaneously addressing and mutating seven genes, which are involved in the fucosylation and xylosylation of plant glycoproteins, and using one vector, it was demonstrated that the CRISPR/Cas9 technology can be efficiently used for multiplex genome editing in the BY2 cells. Note that in order to achieve loss of function, we had to produce bi‐allelic mutations in seven genes; therefore, a total of 14 genetic loci were knocked out. Earlier studies reported on the knockout of multiple loci in polyploid plants (Wang *et al*., 2014). To our knowledge, this is the highest number reported so far in plants.

The glyco‐engineered BY2 cell lines lacking fucose and xylose provide a valuable platform for producing potent biopharmaceutical products that can be similar to the mammalian proteins. The CRISPR/Cas9 technology can be further utilized for knocking out other unwanted genes.

## Experimental procedures

### Plant cell suspensions

*Nicotiana tabacum* cv. BY2 cells (Nagata, 2004) were cultured as a suspension culture in liquid MS‐BY2 medium (Nagata and Kumagai, 1999) at 25 °C with constant agitation on an orbital shaker (85 r.p.m.). The suspensions were grown at 50 mL of volume in 250 mL erlenmeyers and were subcultured weekly at 2.5% (v/v) concentration.

### Isolation and verification of the targeted genes

To isolate fragments of the *XylT* and *FucT* genes from the BY2 cells genome, genomic DNA was extracted from the cells and the appropriate pair of primers (Table S1) were used in a PCR performed according to the PWO DNA polymerase protocol (Roche).

### Construction of the Cas9/sgRNA plasmid and transformation of BY2 cells

Three binary vectors, that is phCas9‐XylT, phCas9‐FucT and phCas9‐XylT‐FucT (Figure 2), were constructed using the pBIN19 backbone vector and were used to transform the tobacco BY2 cells via the Agrobacterium plant transformation procedure (An, 1985). Once a stable transgenic cell suspension was established, it was used for isolating and screening individual cell lines (clones). Establishing of individual cell lines was conducted by using highly diluted aliquots of the transgenic cell suspension and spreading them on solid medium. The cells were allowed to grow until small calli (plant cell mass) developed. Each callus, representing a single clone, was then resuspended in liquid medium and sampled.

### Screening for the knocked‐out lines

Individual lines were screened for the absence of fucose and xylose by extracting their total soluble proteins and loading 10 μg of protein on SDS‐PAGE followed by transfer to a nitrocellulose membrane and a Western blot analysis using anti‐fucose or anti‐xylose antibodies (Agrisera AS07‐268 and Agrisera AS07‐267). Using the fucose antibodies, a direct ELISA was developed to enable a high‐throughput screening. Lines that were assumed to be knocked out for the *FucT* or *XylT* at the screening stage were then sampled for glycan analysis.

### Detection of the Cas9‐induced mutations in the knocked‐out cell lines

Genomic DNA was extracted using the DNeasy plant mini kit (Qiagen). The DNA was amplified by PCR using specific primers for *XylT* and *FucT* genes (Table S3). The PCR products were subcloned into pGEMT vector. For each sample, 36–60 colonies were sequenced and were aligned with the wild‐type target sequences to determine the mutations.

### Transformation of the mutated lines with DNAseI and purification of DNAseI

Using a binary plasmid and the agrobacterium cell transformation procedure (An, 1985), the three mutated cell lines (i.e. the BY2_ΔXT_, BY2_ΔFT_, BY2_ΔXFT_) and nontransformed BY2 cells were transformed with DNAseI (Figure S14). The content and activity of DNaseI in the harvested medium was assessed using indirect sandwich ELISA and methyl green‐based activity (Sinicropi *et al*., 1994) assays, respectively. The DNaseI was purified from the filtered medium using 2 column purification steps and was then used for measuring specific activity, using the methyl green‐based activity assay, and for glycan analyses.

### Glycan analyses

Glycan analysis of the knocked‐out lines versus the wild‐type BY2 cells was done based on the procedure described by Tekoah *et al*. (2004) with slight modifications. Briefly, sample preparation and analysis were as follows: total soluble proteins were extracted from the cells using sample buffer followed by 10‐min boiling of the samples in a water bath. The extracts were centrifuged, and the supernatant was transferred to a clean tube. The total protein concentration of each sample was estimated by dot blot analysis and 200 μg of protein from each sample was then further reduced, alkylated and centrifuged to remove residual precipitates and the supernatant was run on a 12.5% SDS‐PAGE until all loaded samples entered the separation part of the gel, without further separation (a run of about 40 min). For DNaseI samples, 200 μg protein was reduced, alkylated and run on a similar SDS‐PAGE system. After staining with coomassie, the protein bands were excised, cut into small pieces and digested by trypsin. The resulting peptides were extracted from the gel pieces, and the glycans were released by digestion of peptides with PNGase A (an endoglycosidase that releases all types of glycans including α1‐3 fucose). The released glycans were cleaned and fluorescently labelled using 2‐anthranilamide (2AB), followed by removal of the excess 2AB. The labelled glycans were then analysed by separation on a UPLC system using a Waters BEH amide glycan column coupled with a fluorescence detector (Waters, Milford, MA). Dextran hydrolysate was used as a glucose unit (GU) ladder for assignment of the glycans. Further exoglycosydase digestion using a fucosidase and hexosaminidase (Prozyme, CA), followed by HILIC separation, combined with MALDI‐ToF MS analysis was used to confirm the various glycans in the original glycan pool of each sample.

## Author Contribution

Uri Hanania, Tami Ariel and Yoram Tekoah contributed equally to this study, designed the research and were involved in writing the original manuscript and all subsequent revisions.

## Conflict of interest

All of the authors are or were employees of Protalix Biotherapeutics, the sponsor of this study, and as such have vested commercial interests.

## Supporting information


**Figure S1** Sequence of the PCR produced fragment of the BY2‐*XylT‐A* gene.
**Figure S2** Sequence of the PCR produced fragment of the BY2‐*XylT*‐B gene.
**Figure S3** DNA sequence of a fragment of the BY2‐*FucT*‐A and BY2‐*FucT*‐B obtained by PCR.
**Figure S4** DNA sequence of a fragment of the BY2‐*FucT‐*C obtained by PCR.
**Figure S5** DNA sequence of a fragment of the BY2‐*FucT‐*D obtained by PCR.
**Figure S6** DNA sequence of a fragment of the BY2‐*FucT‐*E obtained by PCR.
**Figure S7** The DNA sequence of the tracrRNA backbone.
**Figure S8** Ponceau staining of the membranes that were used for subsequent Western blots.
**Figure S9** Mutations of the *XylT* genes in the ΔXT cell line.
**Figure S10** Mutations of the *FucT* genes in the ΔFT cell line.
**Figure S11** Mutations of the *XylT* and the *FucT* genes in the ΔXFT cell line.
**Figure S12** Exoglycosidase digestion of glycan pool derived from a BY2_ΔXFT_ cell line.
**Figure S13** MALDI‐ToF mass spectrum of the glycan pool derived from a BY2ΔXFT cell line.
**Figure S14** DNaseI protein sequence.
**Figure S15** Exoglycosidase digestion of glycan pool derived from a DNaseI_ΔXFT_ protein.
**Figure S16** MALDI‐ToF mass spectrum of the glycan pool derived from a DNaseI_ΔXFT_ protein.
**Table S1** Primers used for the isolation and amplification of the *XylT* and *FucT* genes from the BY2 cells
**Table S2** Details of the DNA sequences selected as the CAS9 targets (crRNAs)
**Table S3** Primers used for identification and characterization of the mutations in the knocked‐out cell lines
