# Supplementary material for: Establishment of a tobacco BY2 cell line devoid of plant‐specific xylose and fucose as a platform for the production of biotherapeutic proteins
Source: Plant Biotechnol J. 2017 Mar 3;15(9):1120–9. doi: 10.1111/pbi.12702 (PMC5552476; doi:10.1111/pbi.12702)
Supplement: Supplementary file 1 — Figure S1 Sequence of the PCR produced fragment of the BY2‐XylT‐A gene. Figure S2 Sequence of the PCR produced fragment of the BY2‐XylT‐B gene. Figure S3 DNA sequence of a fragment of the BY2‐FucT‐A and BY2‐FucT‐B obtained by PCR. Figure S4 DNA sequence of a fragment of the BY2‐FucT‐C obtained by PCR. Figure S5 DNA sequence of a fragment of the BY2‐FucT‐D obtained by PCR. Figure S6 DNA sequence of a fragment of the BY2‐FucT‐E obtained by PCR. Figure S7 The DNA sequence of the tracrRNA backbone. Figure S8 Ponceau staining of the membranes that were used for subsequent Western blots. Figure S9 Mutations of the XylT genes in the ΔXT cell line. Figure S10 Mutations of the FucT genes in the ΔFT cell line. Figure S11 Mutations of the XylT and the FucT genes in the ΔXFT cell line. Figure S12 Exoglycosidase digestion of glycan pool derived from a BY2ΔXFT cell line. Figure S13 MALDI‐ToF mass spectrum of the glycan pool derived from a BY2ΔXFT cell line. Figure S14 DNaseI protein sequence. Figure S15 Exoglycosidase digestion of glycan pool derived from a DNaseIΔXFT protein. Figure S16 MALDI‐ToF mass spectrum of the glycan pool derived from a DNaseIΔXFT protein. Table S1 Primers used for the isolation and amplification of the XylT and FucT genes from the BY2 cells Table S2 Details of the DNA sequences selected as the CAS9 targets (crRNAs) Table S3 Primers used for identification and characterization of the mutations in the knocked‐out cell lines [file PBI-15-1120-s001.docx]

**Supplementary information**

**Table S1:** Primers used for the isolation and amplification of the *XylT* and *FucT* genes from the BY2 cells. For – forwards; Rev – reverse.

| # | Name | Sequence |
| --- | --- | --- |
| 1 | For-XylT | 5’ ATGAACAAGAAAAAGCTGAAATTTCTTGTTTC 3’ |
| 2 | Rev-XylT | 5’ CTCACAATGGCCATCTACAAAAACC 3’ |
| 3 | For-FucT | 5’ ATGGCAACAGTTATTCCAATTCAAAGGTTACC 3’ |
| 4 | Rev-FucT | 5’ CATTTCCATCCCGGTTACGATGACA 3’ |
| 5 | For-FucT | 5’ GGTTAGTGCTCTTCGTTACATTGAGTCAC 3’ |
| 6 | Rev-FucT | 5’ CATCCAGAAAGATGATTTGTCCACAACATT 3’ |

**Table S2**: Details of the DNA sequences selected as the CAS9 targets (crRNAs)

| # | Name | Targeted sequence | Targeted genes |
| --- | --- | --- | --- |
| 1 | crRNA1 | 5’ GAGAAATTGGAGTCGGTTATTGG 3’ | BY2-XylT-A  BY2-XylT-B |
| 2 | crRNA2 | 5’ GATCGGAATTTGGAAACTGGG 3’ | BY2-FucT-A  BY2-FucT-B  BY2-FucT-C |
| 3 | crRNA3 | 5’ GCTGGCACGGCTAGCGTGCTTCGG 3’ | BY2-FucT-A  BY2-FucT-B  BY2-FucT-C |
| 4 | crRNA4 | 5’ GCCGCTTTCATTTCTAATTGTGG 3’ | BY2-FucT-D  BY2-FucT-E |
| 5 | crRNA5 | 5’ GGGCTTCTAAAGCTTGCAAGAGG 3’ | BY2-FucT-D  BY2-FucT-E |

Targeted nucleotides (black letters), PAM is present at their 3' end (red letters).

**Table S3:** Primers used for identification and characterization of the mutations in the knocked out cell lines. For – forwards; Rev – reverse.

| # | Name | Sequence |
| --- | --- | --- |
| 1 | For-XylT(A,B) | 5’ CTCTTCGCTCTCAACTCAATCACTCTC 3’ |
| 2 | Rev-XylT(A,B) | 5’ ATTAAYTCACGCATAGTGTGCCTTGAAAT 3’ |
| 3 | For-FucT(A,B,C) | 5’ GAAGGTGTTGGGTCATCATCACCTACAAA 3’ |
| 4 | Rev-FucT(A,B,C) | 5’ TTCTAGATGTGCTTACCCACCGTCGTGC 3’ |
| 5 | For-FucT(D,E) | 5’ GGTTAGTGCTCTTCGTTACATTGAGTCAC 3’ |
| 6 | Rev-FucT(D,E) | 5’ CATCCAGAAAGATGATTTGTCCACAACATT 3’ |

1 ATGAACAAGA AAAAGCTGAA ATTTCTTGTT TCTCTCTTCG CTCTCAACTC AATCACTCTC

61 TATCTCTACT TCTCTTCCCA CCCTGATCAC TTCCGCCACA AATCCCGCCA AAACCACTTT

121 TCCTTGTCGG AAAACCGCCA TCATAATTTC CACTCTTCAA TCACTTCTCA ATATTCCAAG

181 CCTTGGCCTA TTTTGCCCTC CTACCTCCCT TGGTCTCAAA ACCCTAATGT TGTTTGGAGA

241 TCGTGCGAGG GTTACTTCGG TAATGGGTTT ACTCTCAAAG TTGACCTTCT CAAAACTTCG

301 CCGGAGTTTC ACCGGAAATT CGGCGAAAAC ACCGTCTCCG GCGACGGCGG ATGGTTTAGG

361 TGTTTTTTCA GTGAGACTTT GCAGAGTTCG ATCTGCGAGG GAGGCGCAAT ACGAATGAAT

421 CCGGACGAGA TTTTGATGTC TCGTGGAGGT GAGAAATTGG AGTCGGTTAT TGGTAGGAGT

481 GAAGATGATG AGCTGCCCGT GTTCAAAAAT GGAGCTTTTC AGATTAAAGT TACTGATAAA

541 CTGAAAATTG GGAAAAAATT AGTGGATGAA AAATTCTTGA ATAAATACTT ACCGGAAGGT

601 GCAATTTCAA GGCACACTAT GCGTGAGTTA ATTGACTCTA TTCAGTTGGT TGGCGCCGAT

661 GATTTTCACT GTTCTGAGGT TAGATTTTGA ATTTTTGTTT GCTCTTTAAA TTAAAGGTTT

721 AAACTTTGTG AATGTTGGCA GATATGGAAT ACACTAATGG ATTTTGTTTG ATCTGTTTAA

781 TGAAGATTGT CTAGAACCTC AATGTTATAA ATATGGTTTG GTTGCTTCAT TAATTAAAGA

841 GAATTCCTTA ATATCCCGAC TAGATGCCAG ATAACACCAG TTAGTTGACT TTTGGATTAT

901 TGGTTGCATT TCATTTGATC AGATAAATTG TTCATTCTTA AATGTTTCAC TAAAGAATTA

961 CTCAAGATTT CAGAGTTTAT ATGTAGGTGT ATGTATTTGG AATTCTGGAT TTGGATCTAG

1021 TATTGAATGG ATTACTGAAC TTGTACTCCC CAGTCATCTG GGGAGGAGCA ATAGATCAAA

1081 TTCAAGGGTT GAAAAGTAAT ACTGAGTCAG AAATTAACCA CTTTAACTTG GAAAACGGTA

1141 AATGTATGTG TTCTAAGATG GTTATTCCTA TAACTTTTGA TGTCTAATAT GGAGAAAGTG

1201 AGTTGATTTA TGCTTTTTCC TTTTCCCTTT ATTGGTGTTG GTTTTTAAAT TCTATCAATT

1261 CCTTTGTTTG ATTGCTACTC AAATTGAACC TTAGACGGAG TAGCAATAGC AAAAAGTGAA

1321 GGCCATTCTT TTCTCCTTTC ATCTCTTTAT TTCCGTTTGA CATACAGAAT ATGGTAGCAT

1381 CTGTCTGAAG TGGTTAATTT TATTCCTTAA AATTTGCATA ACTAATTCGA GTAAATGCCT

1441 TTTGAAGCTT TAGTTGAATA GTTCTACAAC TGGTTGTTGC ATTTTGAGGA CTATCGACTT

1501 GATTTGACAC TTGACATTGT CTGATACATG GCTTGTAAGT TATGAAAACT TTTATCTAGG

1561 AAGAAATCCC AACCAGAGAT AGGGAGCTGT CACTTGGTTA TGAGCTACTG GCTCAAAGTT

1621 CAAGTTTGAC CAGTTAATTT TAGATCTTCA CCAGGATAAC ATTTAGAGTC TAATCAAATT

1681 CTGAAGCAGT ATTGTGCACT AATAAGAGGA ACACATGAAG GATGTAGCAC TACTAGGTTA

1741 TGTTACCTTA TTTACTAATG ATTGACAACC AGCTTAAATG ATGACAAATA GTCTTATATT

1801 TGCTTTTTCA CATTGCTCAT GACTTGGGAT ATTTCCGAAT CAACATATTT TAGTTCTTTA

1861 TGTACTTAAC TACTTATCAA AAAATTATCC CTGCTAGATG TTAGTGTTCA AGCAACCATG

1921 CTAGCTTTTA AGGAAGCTCC TTCTTTGATT CATGCCATCT TTCCGAAATC GATGCCTTAC

1981 GTTACTGTCA TTTTTCTAAT TTTCATTTCA GTGGATTGAG GAGCCGTCAC TTTTGATTAC

2041 ACGATTTGAG TATGCAAACC TTTTCCACAC AGTTACCGAT TGGTATAGTG CATACGTGGC

2101 ATCCAGGGTT ACTGGCTTGC CCAGTCGGCC ACATTTGGTT TTTGTAGATG GCCATTGTGA

2161 G

**Fig. S1:** Sequence of the PCR produced fragment of the BY2-*XylT-A* gene

Black letters represent exons sequences. Exon 1: nucleotides 1-678 and Exon 2: nucleotides 2,012-2,161. Blue letters represent intron sequence. Intron 1: nucleotides 679-2,011.

1 ATGAACAAGA AAAAGCTGAA ATTTCTTGTT TCTCTCTTCG CTCTCAACTC AATCACTCTC

61 TATCTCTACT TCTCTTCCCA CTCTGATCAC TTCCGTCACA AATCCCCCCA AAACCACTTT

121 CCTAATACCC AAAACCACTA TTCCCTGTCG GAAAACCACC ATGATAATTT CCACTCTTCT

181 GTCACTTCCC AATATACCAA GCCTTGGCCA ATTTTGCCCT CCTACCTCCC CTGGTCTCAG

241 AATCCTAATG TTTCTTTGAG ATCGTGCGAG GGTTACTTCG GTAATGGGTT TACTCTCAAA

301 GTTGATCTTC TCAAAACTTC GCCGGAGCTT CACCAGAAAT TCGGCGAAAA CACCGTATCC

361 GGCGACGGCG GATGGTTTAG GTGTTTTTTC AGTGAGACTT TGCAGAGTTC GATTTGCGAG

421 GGAGGTGCTA TACGAATGAA TCCGGACGAG ATTTTGATGT CTCGTGGAGG CGAGAAATTG

481 GAGTCGGTTA TTGGTAGGAG TGAAGATGAT GAGCTGCCCG TGTTCAAAAA TGGAGCTTTT

541 CAGATTAAAG TTACTGATAA ACTGAAAATT GGGAAAAAAT TAGTGGATGA AAAAATCTTG

601 AATAAATACT TACCGGAAGG TGCAATTTCA AGGCACACTA TGCGTGAATT AATTGACTCT

661 ATTCAGTTAG TTGGCGCCGA TGAATTTCAC TGTTCTGAGG TTAGATTTTG AAATTTTGCT

721 TGATCTTTAA ATTAAAGGTT TGAACTTTGT GAATGTTGGC AGATATGGAA TACAATAATG

781 GATTTTGTTT GATCTGTTTA ATGAAGATTG TCTAGAACCT CATTGTTATA AATATGGTTT

841 GTTTGCTTCA TTAATTAAAG AGCATTCCTT AAAATCTCGA CTAGATGCCA GATAACACCA

901 GTTAGTTGAC TTTTGGATTA TGGATTTTTT TTCATTTAAT CAGATAAGAT AGTCATTCTT

961 AAATGTTTCA CTAAAGAATT TGTCATGATT TCAGTGTATA TCTTTAAGTG TATTTGGAAT

1021 TTTGGATTTG GATCTAGTAC TGAATGGGTA ACTGCACTTG TACTCCCCAG TCATCTGGGG

1081 AGGAGCAACA GATTAAATTC AAGGGTTGAA AAGTAATACA GAGTCAGAAA TTAACCACAA

1141 GTTGGAAAAT GGTAAATGTA TGTGGTCTAA GATGATTACT CCTATAACTT TTGATGTCTA

1201 ACATGGAGAA AGTTAGTTGA TTTATGCTCT TTACTTTTCC CTTTATTGAT TTTGGTTTTC

1261 AAATTCTATC AATTCCTTTG TTTGATTGCT ACTCAGATTG AACCTTAGAC GGAGTAGCAA

1321 TAGAAAAGTG AAGAAAAGCC ATTTTTTCTC CTTTCATCTC TTTATTTCTG TTTTACACAC

1381 AGAATATGGT AGCATCTGTC TGAACTAGTT AATTTTATTC CTTAAAATTT GCATAACTAA

1441 TTCGAGTAAA TGCCTTTTGA AGCTTTAGTT GTACAACTGG TTGTTGCATT TTGAGGACTA

1501 TCGACTTGAT TTGACAGTGT CTGATACATG GCTTGTAAGT TATGAAAACT TATATCTAGG

1561 AAGAAATCCC AACCAGAGAT AGGGAGCTGT CGCTTGGTTA TGAGCTACTG GCTCAAAGTT

1621 CGAGTTTGAC CAGTTAATTT TAGATCTTCG GAGTCTAATC AAATTCTGAA GCAGTATTGT

1681 GCACTAATAA GAGGAACACA TCAAGGATGT AGCACTGCCA GGTTATGTTA CCTTATTTAC

1741 TAATGATTGA GAACCAGCTT AAATGATGAC AAATGGTCTT AGATTTGTTT TTTACATTGC

1801 TCATGACTTT GGGATATTTC TGGATCAACA TTTTCCAGTT CTTTATGTAC TTATCAAAAA

1861 ATTATCCCTG CTAGAGCCTA GATGTTAGTG TTCAAGCAAC CATGCTAGCT TTTAAGGAAG

1921 CTCCTTCTTT GATTCATGCC ATCTTTCCGT AATCGATGCC TTACGTTACT GTCATTTTTC

1981 TAATTTTCAT TTCAGTGGAT TGAGGAGCCG TCACTTTTGA TTACACGATT TGAGTATGCA

2041 AACCTTTTCC ACACAGTTAC CGATTGGTAT AGTGCATACG TGGCATCCAG GGTTACTGGC

2101 TTGCCCAGTC GGCCACATTT GGTTTTTGTA GATGGCCATT GTGAG

**Fig. S2:** Sequence of the PCR produced fragment of the BY2-*XylT*-B gene

Black letters represent exons sequences. Exon 1: nucleotides 1-699 and Exon 2: nucleotides 1,996-2,145. Blue letters represent intron sequence. Intron 1: nucleotides 700-1,995.

1 ATGGCAACAG TTATTCCAAT TCAAAGGTTA CCAAGATTTG AAGGTGTTGG GTCATCATCA

61 CCTACAAATG TTCCCCAAAA GAAATGGTCC AATTGGCTAC CTCTAGTAGT TGCACTTGTG

121 GTTATAGTTG AAATTGCATT TCTGGGTCGA CTAGACATGG CTGAAAAAGC CAACCTAGTC

181 AACTCTTGGA CTGACTCATT TTACCAGTTT ACGACGTCGT CTTGGTCAAC CTCCAAAGTG

241 GAAATTAATG AGGCTGGGTT GGCTGTGTTA AGGAGTGGTG AGATTGATCG GAATTTGGAA

301 ACTGGGAGCT GTGAGGAGTG GTTGGAAAGG GAGGATTCTG TGGAGTATTC TAGAGATTTT

361 GACAAAGATC CAATTTTTGT TCATGGCGGC GAAAAGGTGA GATTGTTTCT TGTATATGTT

421 TATTCTTTTT ACTAATAAAT GGGGTGAATA GAGCAGAATG GATATAGAGG ATTCATATAG

481 TTGAACACGA ATAGTTTGGG AAGTTCTATG CACTTAAGGA TGTAATAGTT TTTTTTTTTT

541 TAGTGATGAG TGAAGGGCAG CCTTGACGTA ACCGGTAAAG TTGTTGCCAT GTGAGTCACG

601 GTTTTGAGCC GTGGAAACAA CCTTTTGCAG ACATGCAGGG TAAGGCGGCG TAAAATAGAC

661 CCTTGTGGTC CGGCTCTTCT CGGAACCCTA CATAGCCGGA GCTTAGCGCA TCGACCTGCC

721 ATTTTGATGA TGAGTATTTT GATGATGAGT ATAAGTTCTA TGCACTAACG ATGTAAAATA

781 GATTTACACC ATTAGGTCAC TTAAAAGGTA GTAGCAGCTA ATTCTCCATG GAAACATTAA

841 TTGGTAATCG AGCATCTCTT TTAGACTATT ATATGGATTT TGTTGCAATT TATGTCTTGT

901 TATTTATTAC ATCAGTTAGC TGCCAGAGCT CCAGACATAT GGCTTTGGTA GATGGAATGA

961 TAAAAATTTG TTGTTTCAAA TGATGCTTGG GTTTTCTTCT CGTTTTTTTT TTGTTCTTAT

1021 ATCATGTTTA CAGTGGAATA TCTTATAAAG TACAGACTAT TGACAATATT CCAAAACCTC

1081 TTCTATATGG GTAAAAATAC TCATGAATTT TATATACTAG TAGGGAATAA GGGGAGGAGC

1141 CTTGAGTATC TTCTTTTTTT CTTACCTTTT TTTCCTTCAT TACATTGAAT TCTTCATAGA

1201 GCACTTAAGT GGAATGAGCA GAAATCAATC AGTAAAACTG CCATTTATTG CTTGAGTTTA

1261 TCATGACTAG TTCTTGTTCC CATGTTATCC ACTGGTATAT AGGTGAGAGC AGGTAACAGT

1321 TACTCGGTTA CCATCTTTCT TCTTGATTTT TTTCCCTTAC CATATGCAAA AACTGATGTT

1381 CCTTCGATCG TTATCTAGGA TTGGAAGTCT TGTGCCGTAG GATGTAACTT TGGTGTGGAT

1441 TCTGATAAGA AGCCTGATGC GGCATTTGGG ACACCACAAC AGGCTGGCAC GGCTAGCGTG

1501 CTTCGGTCAA TGGAGTCTGC TCAATACTAT CCTGAGAACA ACATCATTAC CGCACGACGG

1561 TGGGTAAGCA CATCTAGAAA AAGACTTAAA ACATTCTCAC CACATTTGGC AGCATTTGTC

1621 GACAATTGAA TTTTCATCTT GTGATCATTT TTTAATGAAA CATATCTCAC TTGGAAGTTT

1681 TTGCTTGCAA TTAGTTTCTG ACTAGACCTT TTTTCTTTGG ATAAGTAAGG TAGCATATTA

1741 TTAGTAAGCA GTACCAAGAA AGTACAAAAA TTGATACTTC CAAAGTCTAC TCAAAACCTG

1801 AATCCAGCGA CTCCAAGAAC TCATTTGTAC TAACTACAAG ATCCGTTTTA TGCCAGCAAA

1861 AGAGATATTG TAAACATCTA TACTTCATAA CTATAATCTC CTCATTTTCC CCCTCAAAAT

1921 ATCTCATATT TCTTTCTTGC CAAACCGTCA AAACAATACA CAAAGAAATA AGCTTCCAGA

1981 TATTCTTCCC TCTTTTCTCC ACTGTGCTTG CCAGCTGATG AGTGCACCTT TGAAATTTTG

2041 TGGCATTACC CAGTATTACA CCCAAAATAT ATTTTTTATC AGGATACCCT CTAAATATTA

2101 AGGAATAAAG ACCAGATACT CCAAGAAAGA TTACGATGCA TCGGGAGATG ACTAACAGAT

2161 TCACATAGAC AATCCTGATT TGAAACCACA ACTGATCACA GTTGGGAATA AATCTGTAAG

2221 TAAACCTTCA TTACACCATC TATCAGTCCA AAGCTTGACT TTCCTACCAT TTTCAACTTT

2281 TTGTTTTATG TTGTCTTTGA ACTGTCCCTA GAAATTAGCT ATTGGTCTCC CCAAAGCAAC

2341 CTCATAAGGA TTACTTACTG GTTTTGGATC CTAATATCTC TCCATGCCAT AAATTGACTT

2401 AATAACAGCC TTCCATACTG CATATTTTCC ATCGTTACAA AAAAAAGCAG TTGCATTTGC

2461 TCAAATAGCC TTTGGAAAGG GCATCTACAA ACATGCAATC ATAAAGCCCT CAACAACAAT

2521 AACTACAACA ACAACCTAGT AAAATCCCAC AAGTGGGGTC TGGGGAGGGT AGTGTGTACC

2581 CAAACCTTAC CCCTACCCTG AGGGAGTAGA GAGGTTGTTT CCGATAGACC CTCGGCTCAA

2641 GAAGATGAAA AGAGACAATA TATCAGTACT ATCAACAGAT CATAGAGATA ATAACAGCAA

2701 TCATAAAGGC CTCATAGACA CAATAACCTT AGGATCATGT TGTGGTTATA ATTTAATTTT

2761 TAGATCTCCT ATAGTTCTTC TCTCAATCTT TATATCTTTC TCTAGGGAAA TCTCTAACCA

2821 ACTTTATTAT TCTTTCTCAT GTTTCAGAAG GGGATATGAT ATTGTAATGA CAACAAGCCT

2881 CTCTTCGGAT GTTCCTGTTG GGTACTTCTC TTGGGCGGAG TACGATATAA TGGCTCCAGT

2941 GCAACCTAAA ACTGAGAATG CATTAGCAGC TGCTTTTATT TCTAATTGTG GTGCTCGCAA

3001 CTTCCGGTTG CAGGCTCTTG AAGTCCTTGA AAGGGCAAAT ATCAAGATTG ATTCTTTTGG

3061 CAGTTGTCAT CGTAACCGGG ATGGAAATG 3089

**Fig. S3:** DNA sequence of a fragment of the BY2-*FucT*-A and BY2-*FucT*-B obtained by PCR. Black letters represent exons. Exon 1: nucleotides 1-397; Exon 2: nucleotides 1,400-1,559; Exon 3: nucleotides 2,848-3,089. Blue letters represent introns. Intron 1: nucleotides 398-1,399; Intron 2: nucleotides 1,560-2,847.

1 ATGGCAACAG TTATTCCAAT TCAAAGGTTA CCAAGATTTG AAGGTGTTGG GTCATCATCA

61 CCTACAAACG TTCCCCTTAA GAAATGGTCC AATTGGCTAC CTCTAGTAGT TGCACTTGTG

121 GTTATAGTTG AAATTACATT TCTGGGTCGA CTGGACATGG CTGAAAAAGC CAACCTGGTC

181 AACTCTTGGA CTGACTCATT TTACCAGTTT ACGACGTCGT CTTGGTCAAC CTCCAAAGTG

241 GAAATTAGTG AGACTGGGTT GGGTGTGTTG AGGAGTAGTG AGGTTGATCG GAATTTGGAA

301 ACTGGGAGCT GTGAGGAGTG GTTGGAAAAG GAGGATTCTG TGGAGTATTC TAGAGATTTT

361 GACAAAGACC CAATTTTTGT TCATGGCGGC GAAAAGGTGA TATAGTTTTC TTGTAAATGT

421 TTATTTTTTT TTTAGAGCAG AATGAATATA GAGGATTCTT ATAGTTGAAC CCGAACAGTT

481 TAGGAGGCAT AGTTGATTGC TCTTTTTTTG ATGATGCGTA TTAGTTCTAT ACAGGGGATT

541 CTTGGAGTAA CGGTAAAGTT GTCTCCGTAG GTCACAGGTT CGAGCCGTGG GTTCGAGCCG

601 TGGAATCAGC CACGATGCTT GCATCAGGGT AGGCTGCCTA CATTATAACC CCTTCGTGCA

661 CCGGACTACT GCCCTTTAGT ATTAGTTCTA GGCACTAACG ATGCAAAAAA GATTTACGCC

721 ATAAGGTCAC TTAAAAGGTA GTAGCAGCTA ATTCTCCATA GAAACATTAA TTGGTGATCG

781 AGCATCTCTT TTAGACGATA ATTTGAATTT TGTTGCAATT TATGTCTTGC TATTTATTAC

841 ATCAGTTAGC TGCCAGAGCT CGTGTGTGTG TGTGTGTGTG TGTGTGTGTT CCAGACTTGT

901 GGCTTTGGTA GGTGGAATGA TAAAAAAATT GTTGTTTCAA ATGATGCTTT GGCATTTTCT

961 TCTAGCTTTT TTGTTCTTTT ATCATGTTTA CGGTCAAACA TCTTATAAGG ACTATTGACC

1021 ATATTCCAAA ACCGATTATA TGGGTAAAAA TACTCATGAA TTTTATATAC TAGTAGGGAA

1081 TAAGTGGAGG AGCCTTGAAT ATCTTCTTTT TTCTTACCTT TTTTTCCTTC ATTACATTGA

1141 ATTCTTCATA GAGCACTTAA ATGGAATGAG CAGAAATTAA TCAGTAAAAC TGCCATTTAT

1201 TGCTTAAGTT TATCATGAGT AGTTCTTGTT CCCATGTTAT CCACTGGCAT GTAGTGAGAG

1261 CAGGTAACAG TTACCTGGTT ACCATCTTTC TTCTTGATTT TTTTTTCCTT ACCATATGCG

1321 AAAACTGATG TTCCTTCAAT CATTATCTAG GATTGGAAGT CTTGTGCCGT AGGATGTAAC

1381 TTTGGTGTGG ATTCTGAAAA GAAGCCTGAT GCGGCATTTG GGACACCACA ACAGGCTGGC

1441 ACGGCTAGCG TGCTTCGGTC AATGGAGTCA GCTCAATACT ATCCTGAGAA CAACATCGTT

1501 ATGGCACGAC GGTGGGTAAG CACATCTTGA AAAAGATTTA AAACATTCTC ACCTACATTT

1561 GGCACCTGAA AGATAATAGC ATTTGCCACA TTTGAGTTTT CATCTTGTGA TCGTATCTTA

1621 ATCAAACATA TCTCACTTGG AAGTTTTTGC TTGCTATTAG TTTCTGACTA GGCCTTTCTT

1681 TTAAGGAGCA TATTATTAGT AAGCAGTACC AAGAAGGTAC CAAAAAAGTA CAAAAATTTA

1741 GACTTCCAAA GACAACTCAA AACCCGAATC TAGTGAGTTG AAGAACTCAT TTGTACTAAC

1801 TAGAGGATCG GTTTTATGCC AGCAAAAGAG ATACTGTAAA CATCTATTCT TTATAACTGA

1861 ATTATGCTCC TTTCCCCCCT CAAAATATCT CGTGTTTCTT TCTTGACAAA ATCTCCAAAT

1921 AATACACAAA GGAATAAGCT TGCAGATCTT CTTCAGTCTT TTCTCCACTG TGCTTGCCAG

1981 CTGATGAGTC CACCTTTGAA ATTTGTGGCA TTACCCAGTA TTACCCCCAA AATATATTTT

2041 TTATCAGTAT ACCCTCAAAA TATTAAGGAA TAAAGACCAG CTACTCCAAG AAAGATTACG

2101 ATGCATCAGG AGATGACTAA CAGATTCACA GACAATCCTG ATTTGGAACC ACACTGAACA

2161 CGGCTGAGAA TAAATCTATA AGTAAACCTT CATTACACTA TTCATCACTC CAAAGCTTGA

2221 CTTTCCCATC ATTTTCAGCT TTTAGTTTTA TGTTTATCTT TGAACTGCCT CCAAAAATTA

2281 GCTATTGGTC TCCACAAGCA ACCCATAAGG ATTACTTACT GGTTTTGGAT TCTAATATCT

2341 CTCCATGCCA TAAATCGGCT TTATAACAGC CTTCCATACT GCATATTTTC TATCATTACG

2401 AAAAAAAAGC AGTTGCATTT GCTCAAATAG CCTTTGGAAA GGGCATCTAC AAATATGCAA

2461 TCATAAAGCC CTCATAGACA ATAATCTTAG GATCATGTTT TTTTTTATGA TAATGGTAAC

2521 ATATATATTA ATAAATAAAA CAGCACTAGA GCAGTGCCAA GCCATATTTA CAAGAAAACA

2581 GAAACCAGCT ATGACAACCT AAGATTACAG TGCACCTATC AGGTCTACTA AAGATTCTGC

2641 CTCCTCTATC CAGTTTTCTT TACACCAGAA ATAAAACAAA AGTAAACACT TCATCTTGAT

2701 GTTCTGTAAA GAATTGCTTC TGTCTTCAAA AGTTCTTAAA TTTCTCTCCT TCCAGACTGT

2761 CCACCATATG CACGCAGGAA CCAATCGCCA CCATTTCTTC TGTCTCACTA TACCCTCATT

2821 GTAATTCCAA CACTTCAACA TATCACAAGT GTTAGCTGGC ATGCTCCATT TAAGGCCCAC

2881 AATATTAAGA AATAGTTGCC ACAGTTGGTC AGTAAAAGGA CAATGAAGAA ACAAATGGCT

2941 ATTAGTTTCT ATAGCTGTAC CACATAATAG GCATTTAGAG CATAGCTGAA AACCTCTTCT

3001 TCTCAAGTTC TCTTGGGTTA GACAAGCCTT TCTTGTAACT AACCATGAAA AGCATACCAC

3061 CTTGAAAGGT GCCTTTACTT TCCAAATATT CCTCCAGGGC CATTGTTCTA ACTGTTGGTT

3121 TGAAGCAGCG AGTAGAGTAT ACGCGGATCT GACCGTAAAA TTCCCACTCC TTCAATGATT

3181 CCAGCATAGT TTATCTTCAG TTTCTCCAAG ACCTTGGAAT TGTTCCAAGA TATTAAAGAA

3241 ATTTACAACC CTTTCTAATT CCCAATCATT CAAGGCCTTT CTAAAAGTAA CATTCCATCC

3301 TTGCTGACCC CAGGCAGTGT CTAAAGTGGA TGTAGGTGAT GTTGCAATGC TGAAAAATTT

3361 TTGAAAAAAT TCTTTGAGAG GACCATGTCC TAACCAGTTA TCACTCCAGA AAAGTGTTTT

3421 CCTTCCATTA CCAACCTTTA TGGTTGTATT ATCAGCCAGA GTATTCCAGT GAAGTCTGAT

3481 TGACTTCCAT ACCCCAACCC CATGTGAACT ATTAACAGAG TTAGAGCACC ATAGTCCATT

3541 TTGTCCATAC TTGTCACAGA TGACCTTTCT CCACAAAGCA TTAGCCTTTC AGATTGTACC

3601 TCCAAAGCCA CTTCAAGAGT AAACTCTGAT TGTGGGCTTT AAGGTTCCTA ATGCCCAAAC

3661 CCCCTTTGTC TTTGCTTGTG ATAAGGGAAT TCCAGTTAAC TAAATGAATA GCTTCCTTCT

3721 CTCTGTTTCC TTGCCATATA AAGCTCCTTC TTAGTGCATC AATTCTTTTC CTCACCTTGC

3781 TTGGTAAGGG AAACAAAGAC ATTACACATG TAGGAAGTGC ATCCAAGACA CTATTAACCA

3841 ATACCACCCT TCCACCCAAG GATAAATACT GGCTCTTCCA GGTTGATAGC CTTTTTTCAC

3901 ATCTTTCAAG AACCCCATTC CATATTTCTT GAGCTTTGTT CTTGGAGCCT AGAGATAAAT

3961 CCAAATATAT AGTTGGTAGA GAACCCACCT CATATCCCAG TATAGCTGCT AGGGCCTGGA

4021 TATCATCCAC TTCTTTAACT GGGAACAACA TGCTCTTCCT CCGATTTACA TGGAGCCCTG

4081 AGACTGCTTC AAATATAACA AGAATGACTC TTAGATATTT TAGTTGATCT GCCTTTGCCT

4141 CACAAAAAAC TAAAGAGTCA TCAGCATATA GAAGATGGGT GATCTCCACA CTATCTCCCT

4201 CCCTGTTTGA AGCTTTAAAA CCTTTCAGCC AACCATTGTT GTTTGCATTT TTTAACATTT

4261 GGTTCAATCC TTCCATAGCA AAGAGGAAAA GAAAAGGTGA TAAGGGATCC CCTTGTCTCA

4321 AACCTCTCTC AGAAGGAAAA AATCCCTCTG GAGAACCATT CACCAAAATG GAAAATCTGA

4381 CTGTTCTGAT GCAGAAAGAT ATCCAGTTGA TCCACTTCCT ACCAAAACCC ATATCCTGAA

4441 GAACTTTGAG TAAAAAGTTC CAATTAACAT GATCATAGGC CTTCTCAATA TCAAGCTTAC

4501 AGAGAATTCC GGGAACCTGC CCTTTTAAGT CAGGAATCCA CACATTCATT TGCAATCAAG

4561 GAAGCATCCA TGATTTGCCT GCCTCTTATG AAGGCCATTT GATGCCCATT CACTAGTTTG

4621 TCCACCACTT TCTTGAGCCT CTCTGTCAAT AGTTTGGAAA TGATCTTGTA AATGCTTCCT

4681 GTAAGACTAA TTGGTCTGAA GTCCTTGAGT TCCTTAGCAC CATTCTTTTT TGGAATAAGG

4741 GCTATGTAAG TAGCATTGAA ACTCTTCTCA AAATATTCAT TGTTGTGGAA GTTTCTGATG

4801 GTATGCATGA TGTCTTCTTT CACAATCTCC CAGCAATTAT GGAAAAACCC CATAGAGAAA

4861 CCATCAGGGC CCGGTGCTTT GTCAGCTGCA CATATCTTCA AGGTCTCAAA CACTTCTTGT

4921 TCTTCAAATT CTCCTTGTAG CAATTCATTA TCTTCCTATA GTTCTCCTAT AGTTCTAATT

4981 GTCTCTCAAT CTTTATATCT TTAAATTTAA ATTTTAGATC TCCTATAGTT CTTCTCTCAA

5041 TCTTTATATC TTTCTCTAGG GAAATCTCTA ACCAACTTTA TTATTCTTTC TCATGTTTCA

5101 GAAGGGGATA TGATATTGTA ATGACAACAA GCCTCTCTTC GGATGTTCCT GTTGGGTACT

5161 TCTCTTGGGC GGAGTATGAT ATAATGGCTC CAGTGCAACC TAAAACTGAG AATGCGTTAG

5221 CAGCTGCTTT TATTTCTAAT TGTGGTGCTC GCAACTTCCG GTTACAGGCT CTTGAAGTCC

5281 TTGAAAGGGC AAATATCAAG ATTGATTCTT TTGGCAGTTG TCATCGTAAC CGGGATGGAA

5341 ATG 5343

**Fig. S4:** DNA sequence of a fragment of the BY2-*FucT-*C obtained by PCR

Black letters represent exons. Exon 1: nucleotides 1-397; Exon 2: nucleotides 1,352-1,511; Exon 3: nucleotides 5,102-5,343. Blue letters represent introns. Intron 1: nucleotides 398-1,351; Intron 2: nucleotides 1,512-5,101.

1 GGTTAGTGCT CTTCGTTACA TTGAGTCACA CGATTAATTT ATGAAGCCAA AGTTCTTAAG

61 GACCATGGCG TGGTTGAGAT TTAAACTTTG CATAAGCTTG CTAACCAATT TTATTTTTTT

121 CACTCACATA CCAGAAGGGG ATATGATGTT GTAATGACAA CAAGCTTCTC TTCAGATGTT

181 CCTGTTGGAT ACTTCTCTTG GGCTGAGTAT GATATCATGG CTCCAGTACA ACCTAAAACA

241 GAGAATGTCT TAGCAGCCGC TTTCATTTCT AATTGTGGTG CTCGCAATTT CCGCTTGCAA

301 GCTTTAGAAG CCCTTGAAAG GGCAAATATC AGAATTGATT CTTATGGCAG TTGTCATCAT

361 AACAGGGATG GAAGAGGTTA GTATATTTCA ATTATCCAAA CTTACTGAAG GATTAGAGGA

421 TAGAATACGG ATGGTGCAAT TTTAAGCAGT GTCACTAGGG AGCTAATTCT TGTCCATAGA

481 GTAGTATTAT GGGTTTGATT GACTCTTCCT CGGGTATCAC ACCTTCCTCC AGAAGACAGG

541 ATTTTACTAC CAGTGCAAAC CTTTTTTTTC TCTCCTGGCT AATGTGAGCA CGCATGTCGT

601 CGTTTTTTTA GTGATTTGAA TTTATGCTAG TCCAATGATT GCTTGTCAAT GGATTATTTT

661 GCTCTTTTTC TTGTTTAAAA TTTGAGTTTC AATTTTGCCA CCTGATAAGA ATAAAGTTGG

721 AATACAACAT TCATTTAAAT AGTTCGATTT CATTCTGAGG AAGTTAGGCT GAGATTTGTT

781 GGAAAGAGAC GTATAGCGAG AAAAAATGTT GTGGACAAAT CATCTTTCTG GATG 834

**Fig. S5:** DNA sequence of a fragment of the BY2-*FucT-*D obtained by PCR

Blue letters represent introns. Partial intron 2: nucleotides 1-134; Partial intron 3: nucleotides 377-834 Black letters represent exons. Exon 3: nucleotides 135-376.

1 GGTTAGTGCT CTTCGTTACA TTGAGTCACA AGATTAATTT ATGAAGCCAA AGTTCTTAAG

61 GACCATGGCG TGGTTGAGAT TTAAACTTTG CATAAGCTTG CTAACCTATT TTATTTTTTC

121 CACTCACATA CCAGAAGGGG ATATGATGTT GTAATGACAA CAAGCCTCTC TTCAGATGTT

181 CCTGTTGGAT ATTTCTCTTG GGCTGAGTAT GATATCATGG CTCCAGTACA ACCTAAAACA

241 GAGAATGCCT TAGCAGCCGC TTTCATTTCT AATTGTGGTG CTCGCAACTT CCGCTTGCAA

301 GCTTTAGAAG CCCTTGAAAG GGCAAATATC AGAATTGATT CTTATGGCAG TTGTCATCAT

361 AACAGGGATG GAAGAGGTTA GTATATCTCA ATTATCCAAA CTTACTGAAG GATTAGAGGA

421 TAGAATATGG ATGGTGCATT TTTAAGCAGT GCCACTAGGG AGCTAATTCT TGTCCATAGA

481 GTAGTATTAT GGATTTGATT GACTCTTCCT CGGGTGTCAC ACCTTCCTCC AGAAGACAGG

541 ATTCTACTAC CAGTGCAAAC CTTATTTTTT TTCTCCTGGC TAACGTGAGC ATGCATGTCG

601 TTTTTTTAGT GATTCGAATT TATGCTAGTC CGATGATTGC TTGTCAATGG ATTATTTTGC

661 TCTTTTTCTT GTTTAAAATT TGAGTTTCAA TTATGCCACC TGATAAGAAT AAAGTTGGAA

721 TACAACATTC ATTTAAGTAG TTCGATTTCA TTCTGAGGAA GTCAGGCTGA AATTTGTTGG

781 AAAGAGACGT ATAGTGAGAA AAAATGTTGT GGACAAATCA TCTTTCTGGA TG 832

**Fig. S6:** DNA sequence of a fragment of the BY2-*FucT-*E obtained by PCR

DNA sequence of BY2-*FucT-*E fragment obtained by PCR. Partial intron 2 is highlighted in red, nucleotides 1-134. Exon 3 is highlighted in turquoise, nucleotides 135-376. Partial intron 3 is highlighted in red, nucleotides 377-832.

GTTTTAGAGCTAGAAATAGCAAGTTAAAATAAGGCTAGTCCGTTATCAACTTGAAAAAGTGGCACCGAGTCGGTGCTTTTTTT

**Fig. S7:** The DNA sequence of the tracrRNA backbone


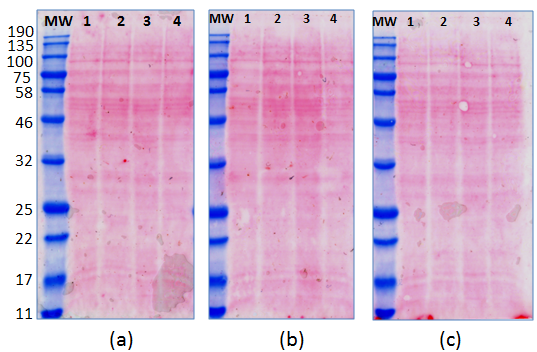


**Fig. S8:** Ponceau staining of the membranes that were used for subsequent western blots

The total soluble protein was extracted from the glyco-engineered and the non-transgenic BY2 cells and 10 µg of protein from each sample were loaded on SDS-PAGE followed by Ponceau staining and subsequent western blots (WB). (a) membrane used for the anti-xylose WB, (b) membrane used for the anti-fucose WB and (c) membrane used for the anti-HRP WB. 1 – ΔXT cell line; 2 – ΔFT cell line; 3 – ΔXFT cell line; 4 – the wild-type non-transgenic BY2 cells. MW - molecular weight marker in kDa.


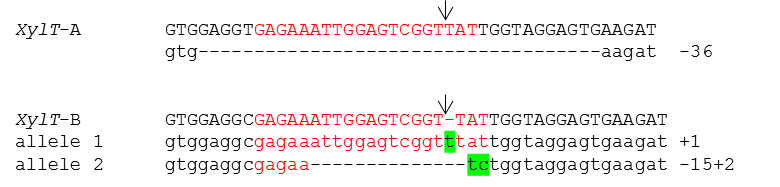


**Fig. S9:** Mutations of the *XylT* genes in the ΔXT cell line

Recognition sequences of the *XylT*-A and the *XylT*-B genes are designated in uppercase and sequences of the mutated alleles are shown below in lowercase. One common target site within exon 1, indicated in red, was designed for *XylT*-A and *XylT*-B. Deletions are indicated by dashes and insertions are highlighted in green. The arrow indicates the precise cleavage site. The size of in-del is shown on the right in bp.


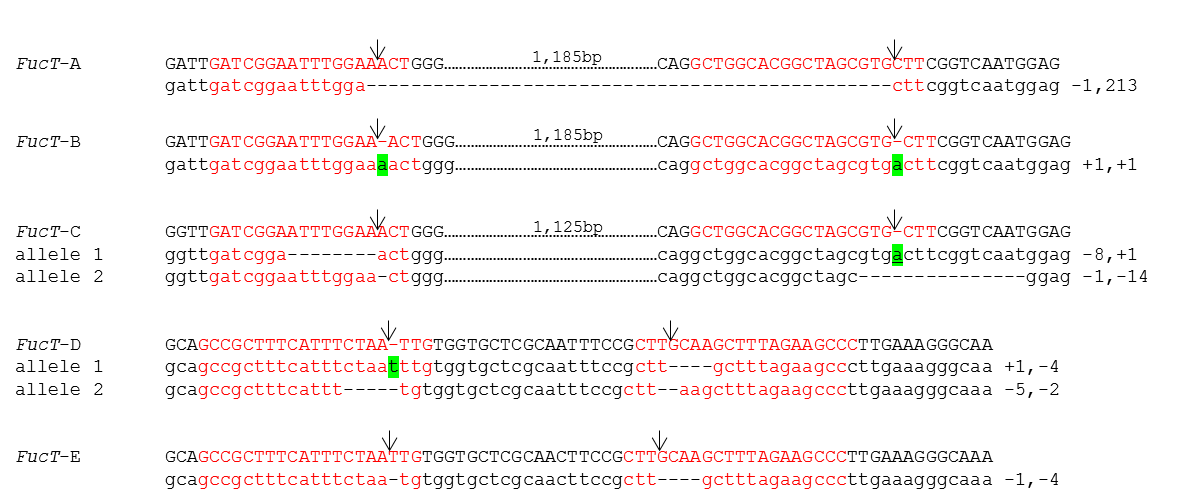


**Fig. S10**: Mutations of the *FucT* genes in the ΔFT cell line

Recognition sequences of the five *FucT* genes are designated in uppercase and sequences of the mutated alleles are shown below in lowercase. Two common target sites within exon 1 and exon 2, indicated in red, were designed for *FucT*–A, *FucT*–B and *FucT*–C. Two common target sites within exon 3, indicated in red, were designed for *FucT*–D and *FucT*–E. Deletions are indicated by dashes and insertions are highlighted in green. The arrow indicates the precise cleavage site. The size of in-del is shown on the right in bp.


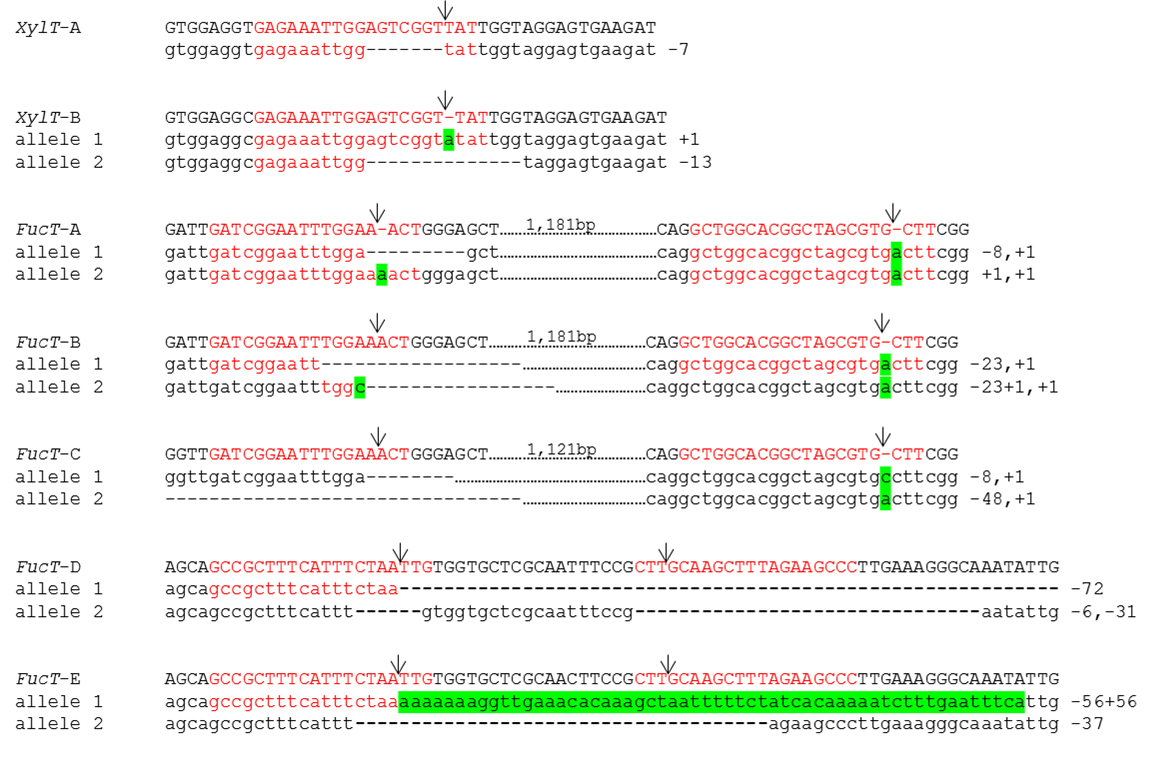


**Fig. S11**: Mutations of the *XylT* and the *FucT* genes in the ΔXFT cell line

Recognition sequences of the *XylT* and *FucT* genes are designated in uppercase and sequences of mutated alleles are shown below in lowercase. One common target site within exon 1, indicated in red, was designed for *XylT*-A and *XylT*-B. Two common target sites within exon 1 and exon 2, indicated in red, were designed for *FucT*–A, *FucT*–B and *FucT*–C. Two common target sites within exon 3, indicated in red, were designed for *FucT*–D and *FucT*–E. Deletions are indicated by dashes and insertions are highlighted in green. The arrow indicates the precise cleavage site. The size of in-del is shown on the right in bp.


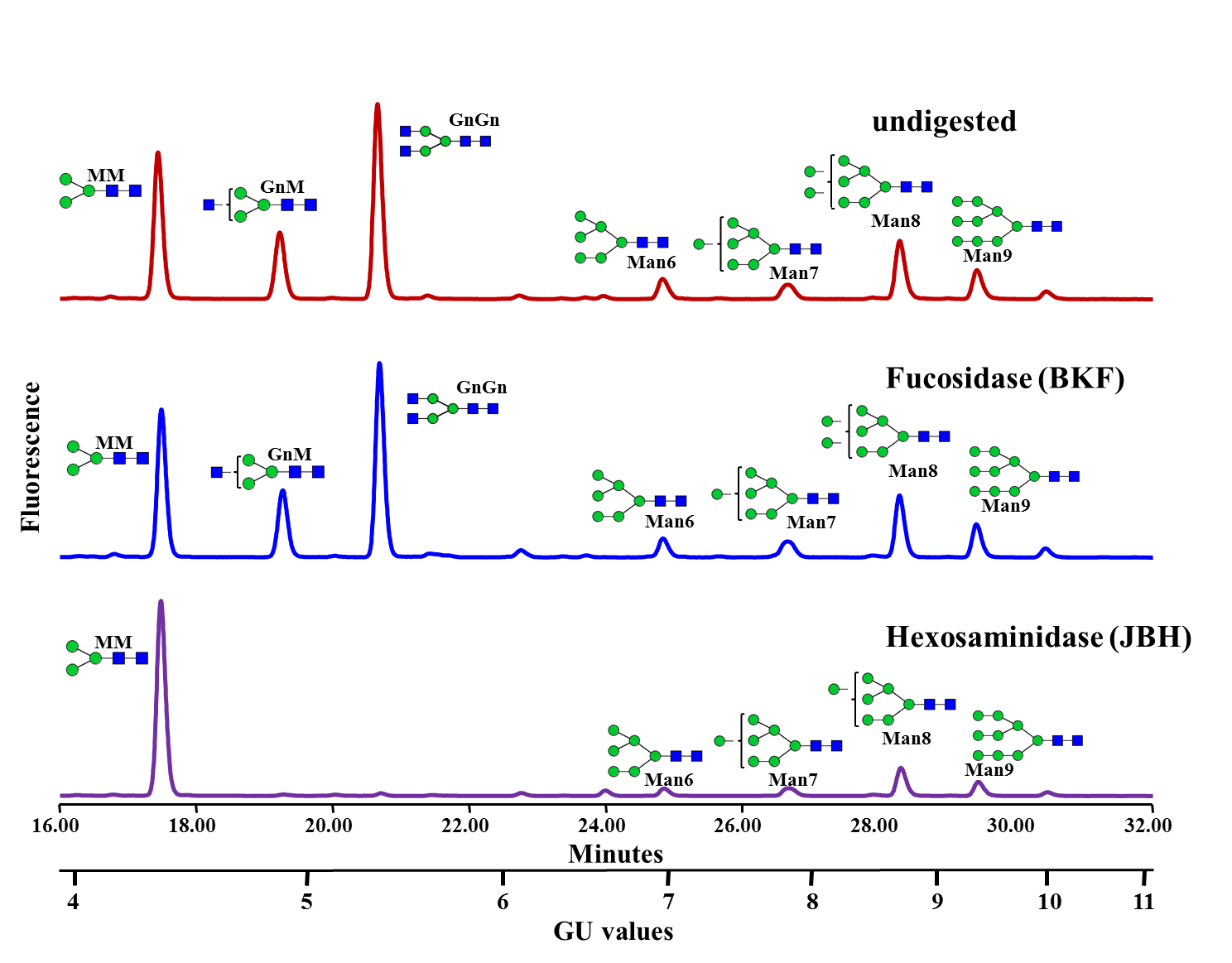


**Fig. S12**: Exoglycosidase digestion of glycan pool derived from a BY2_ΔXFT_ cell line.

Top chromatogram is a separation of undigested glycans on UPLC, showing the original distribution of the glycan pool. Middle panel shows the same pool after digestion with fucosidase, showing the glycans that remain after the removal of fucose. There is no shift in the profile, indicating that no glycans contain fucose. Bottom panel shows the same pool after digestion with hexosaminidase, which releases either one or two terminal N-acetylglucosamine (GlcNAc) from the glycans. The three first peaks combine to a paucimannose structure that has no xylose or fucose. The high mannose glycan peaks do not show any shift, since they do not contain either fucose or terminal GlcNAc.


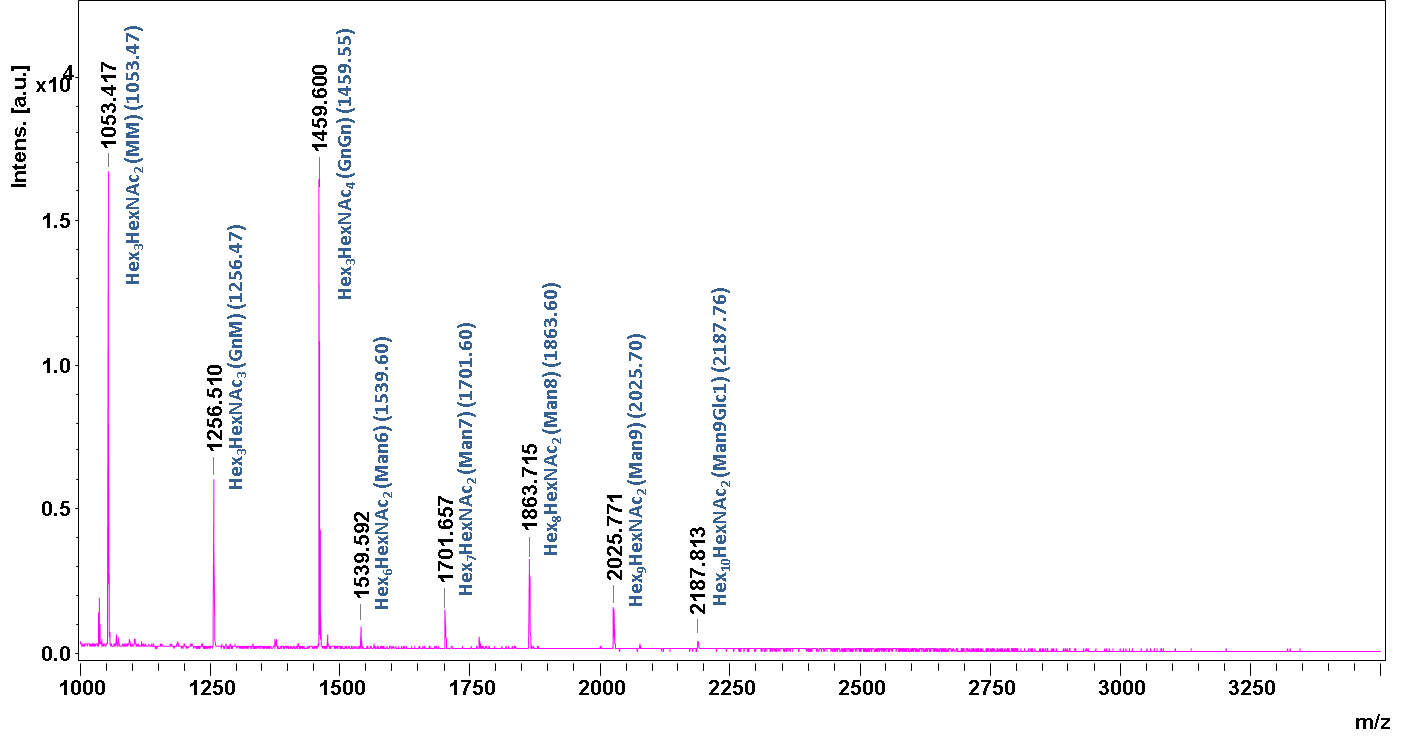


**Fig. S13:** MALDI-ToF mass spectrum of the glycan pool derived from a BY2_ΔXFT_ cell line.

Y axis represents the mass to charge ratio of the ions measured. Major peaks are annotated with the apparent composition of the glycans. Parentheses indicate acronym of each glycan and expected mass values of the corresponding composition.

1 MIVLSVGSASSSPIVVVFSVALLLFYFSETSLGLKIAAFNIQTFGETKMSNATLVSYIVQILSRYDIALVQEVRDSHLTA 80

81 VGKLLDNLNQDAPDTYHYVVSEPLGRNSYKERYLFVYRPDQVSAVDSYYYDDGCEPCGNDTFNREPAIVRFFSRFTEVRE 160

161 FAIVPLHAAPGDAVAEIDALYDVYLDVQEKWGLEDVMLMGDFNAGCSYVRPSQWSSIRLWTSPTFQWLIPDSADTTATPT 240

241 HCAYDRIVVAGMLLRGAVVPDSALPFNFQAAYGLSDQLAQAISDHYPVEVMLK 293

**Fig. S14:** DNaseI protein sequence

The amino acids sequence of the human DNaseI (GenBank: NM_005223, blue letters) fused with the Arabidopsis ABPI leader peptide (red letters). For expression in the BY2 cells the codon usage of the *DNaseI* gene was adapted to the codon bias of *N. tabacum*. The optimized gene was synthesized by GENEART AG (Regensburg, Germany) and was cloned into an appropriate expression vector.


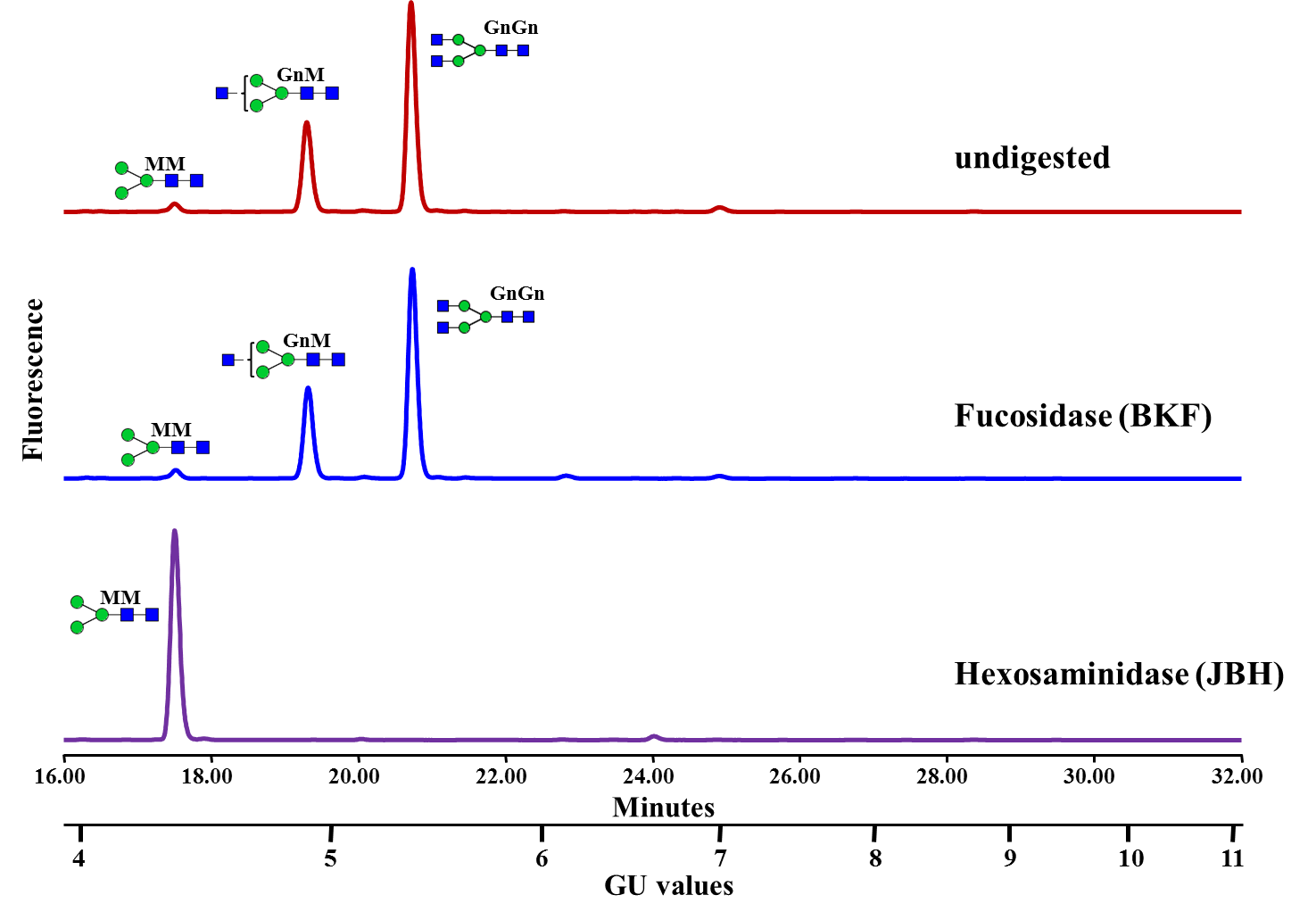


**Fig. S15**: Exoglycosidase digestion of glycan pool derived from a DNaseI_ΔXFT_ protein.

Top chromatogram is a separation of undigested glycans on UPLC, showing the original distribution of the glycan pool. Middle panel shows the same pool after digestion with fucosidase, showing the glycans that remain after the removal of fucose. There is no shift in the profile, indicating that no glycans contain fucose. Bottom panel shows the same pool after digestion with hexosaminidase, which releases either one or two terminal N-acetylglucosamine (GlcNAc) from the glycans. The three first peaks combine to a paucimannose structure that has no xylose or fucose. No High mannose structures were identified.


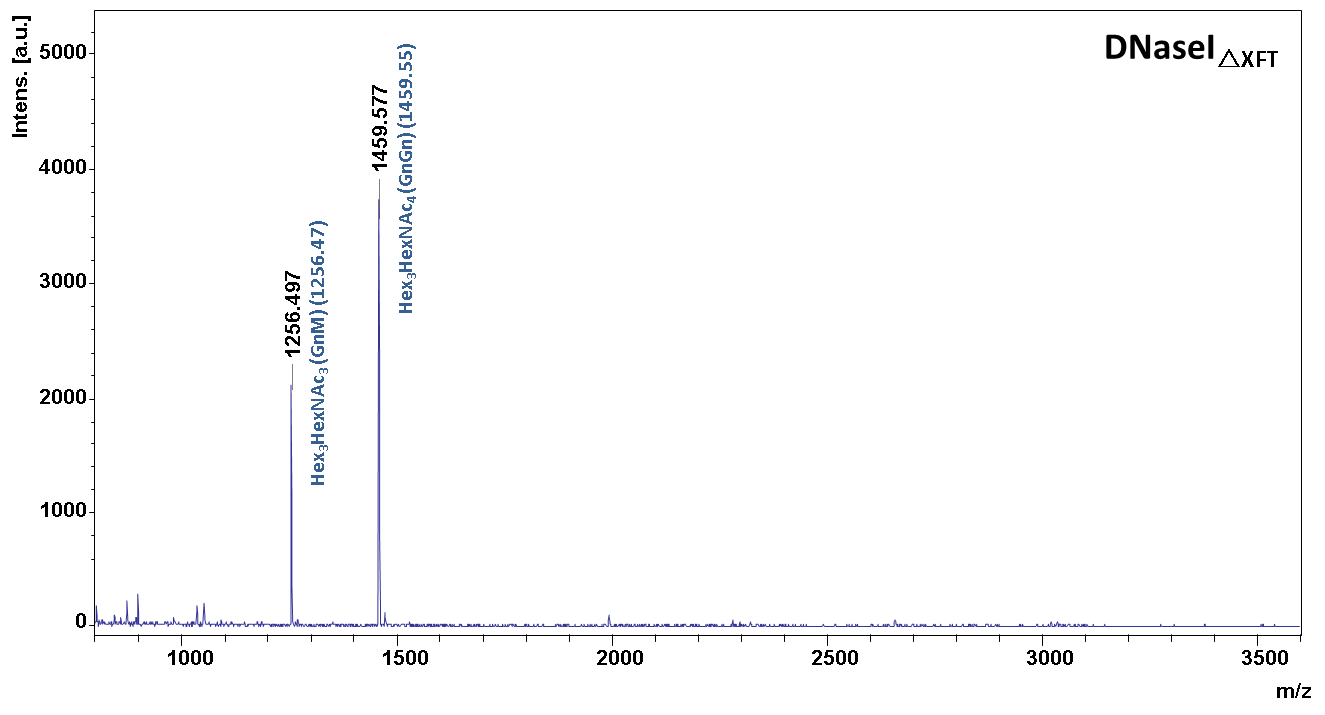


**Fig. S16:** MALDI-ToF mass spectrum of the glycan pool derived from a DNaseI_ΔXFT_ protein.

Y axis represents the mass to charge ratio of the ions measured. Major peaks are annotated with the apparent composition of the glycans. Parentheses indicate acronym of each glycan and expected mass values of the corresponding composition.
